# Frail or hale: Skeletal frailty indices in Medieval London skeletons

**DOI:** 10.1371/journal.pone.0176025

**Published:** 2017-05-03

**Authors:** Kathryn E. Marklein, Douglas E. Crews

**Affiliations:** 1 Department of Anthropology, Ohio State University, Columbus, Ohio, United States of America; 2 College of Public Health, Ohio State University, Columbus, Ohio, United States of America; Museo delle Civiltà, ITALY

## Abstract

To broaden bioarchaeological applicability of skeletal frailty indices (SFIs) and increase sample size, we propose indices with fewer biomarkers (2–11 non-metric biomarkers) and compare these reduced biomarker SFIs to the original metric/non-metric 13-biomarker SFI. From the 2-11-biomarker SFIs, we choose the index with the fewest biomarkers (6-biomarker SFI), which still maintains the statistical robusticity of a 13-biomarker SFI, and apply this index to the same Medieval monastic and nonmonastic populations, albeit with an increased sample size. For this increased monastic and nonmonastic sample, we also propose and implement a 4-biomarker SFI, comprised of biomarkers from each of four stressor categories, and compare these SFI distributions with those of the non-metric biomarker SFIs. From the Museum of London WORD database, we tabulate multiple SFIs (2- to 13-biomarkers) for Medieval monastic and nonmonastic samples (N = 134). We evaluate associations between these ten non-metric SFIs and the 13-biomarker SFI using Spearman’s correlation coefficients. Subsequently, we test non-metric 6-biomarker and 4-biomarker SFI distributions for associations with cemetery, age, and sex using Analysis of Variance/Covariance (ANOVA/ANCOVA) on larger samples from the monastic and nonmonastic cemeteries (N = 517). For Medieval samples, Spearman’s correlation coefficients show a significant association between the 13-biomarker SFI and all non-metric SFIs. Utilizing a 6-biomarker and parsimonious 4-biomarker SFI, we increase the nonmonastic and monastic samples and demonstrate significant lifestyle and sex differences in frailty that were not observed in the original, smaller sample. Results from the 6-biomarker and parsimonious 4-biomarker SFIs generally indicate similarities in means, explained variation (R^2^), and associated P-values (ANOVA/ANCOVA) within and between nonmonastic and monastic samples. We show that non-metric reduced biomarker SFIs provide alternative indices for application to other bioarchaeological collections. These findings suggest that a SFI, comprised of six or more non-metric biomarkers available for the specific sample, may have greater applicability than, but comparable statistical characteristics to, the originally proposed 13-biomarker SFI.

## Introduction

In this paper, we apply a recently proposed methodology for assessing frailty using a skeletal frailty index (SFI, see [1]) and discuss its applicability to archaeological samples. We developed the SFI to assess frailty in skeletal materials following extensive observations on biomarkers of frailty in modern living humans [2–7]. Originally, we proposed a SFI including 13 skeletal biomarkers observable on human skeletons, representing four categories of stressors: trauma, nutrition/disease, physical activity, and growth disruptions. The strength we advocated for in this index was its broad range of measurable and observable biomarkers that capture aspects of both childhood and adult frailty.

Unfortunately, in many skeletal assemblages, available materials are not sufficient to assess all 13 biomarkers included in the original SFI. Using identical samples from Medieval London [1], we examine how versions of the SFI constructed of fewer biomarkers may increase available sample size while maintaining results comparable to the more comprehensive 13-variable SFI. We expect reducing the number of contributing assessments will substantially increase available sample sizes. Our goal is to determine the fewest number of biomarkers needed in a SFI to reproduce results in the Medieval sample equivalent to the original 13-variable SFI, i.e., the lower limits for a robust SFI with statistical similarities in explained variance and significance. Our aim is to balance the maximization of frailty information (number of biomarkers) with population representation (sample size). After exploring applicability and reliability of reduced-variable SFIs (<13 biomarkers) we assess two modified SFIs (4- and 6-biomarkers) for associations with lifestyle (determined by cemetery/location), age, and sex in a larger monastic and nonmonastic Medieval sample than assessed previously to develop the original SFI.

### Skeletal frailty in archaeological samples

Since its inception, bioarchaeology has sought to accurately assess “stress” and “health” among past human populations to address questions in biocultural history [8–10]. Multiple skeletal measures and features have been proposed as estimators of somatic stress and chronic physiological damage, reflections of conditions that challenged the deceased during life, and were preserved in their skeletal and dental tissues [11–16]. Such skeletal biomarkers, in turn, have been used to quantify and establish both direct and indirect interpersonal and interpopulational interactions in the past, based upon their impacts on biological health differentials identifiable in skeletal remains. In living populations, sequelae of life-long stressors frequently are assessed by established frailty indices to identify the frailty phenotype [2, 4, 7, 17], a concept that we recently applied to skeletal samples [1].

Here, we define skeletal frailty using indices based upon criteria applied widely to assess frailty in clinical practice, hospitals, and biomedical research [2–4, 7, 17–22]. Specifically, we use a human biology/physiology framework to define frailty as a phenotype that develops secondary to loss of muscle (sarcopenia) and bone (osteopenia), leading to weakness and reduced physical activity. In human biology, frailty is a distinctive phenotype apart from morbidity and mortality risk, although frailty may correlate with both [7]. While recent definitions of frailty in bioarchaeology research focus on and operationalize frailty as increased risk of mortality [23–25], the SFI places frailty within the theoretical and applied contexts of epidemiology, clinical medicine, and human biology, defining frailty as a fluctuating somatic state of cumulative functional losses that increases with age among the living, relatively independent of mortality risk [7, 17, 26]. This definition of skeletal frailty *may* appear to run counter to the definition of selective frailty, whereby frailty “refers to individual biological characteristics associated with…susceptibility, propensity, or relative risk with respect to disease and death” [27], but initial results from the 13-biomarker SFI support Wood and colleagues’ hypothesis that individuals more susceptible to life’s stressors tend to die before evidence of wear-and-tear skeletonizes [1]. Skeletal frailty, by this consideration, is a reflection of physical decrepitude that cumulates over years, and this frailty paradoxically correlates with lower risk of mortality [27]. Those who survive to older years tend to accumulate more skeletal biomarkers of stress, manifesting greater skeletal frailty than their contemporaries who died at earlier ages [1], reflecting results similar to the male-female morbidity-mortality paradox and the osteological paradox observed frequently in human and nonhuman samples [27–29]. These differences in trajectories of frailty, senescent biology, and mortality between men and women are reflected oftentimes in greater debilitation, but longer life spans, of the latter: women generally outlive men, and they also tend to report more chronic illnesses and disability over their lifespans [7, 28], although exceptions to this pattern have been observed in living and archaeological populations [30–32].

### Skeletal frailty index

The original skeletal frailty index (SFI) was composed of 13 metric and non-metric biomarkers associated with chronic and cumulative stress in living human populations, biomarkers consistently employed and implemented in bioarchaeological research [33]. The SFI builds upon previous works, which have outlined components for archaeological health profiles or have coded and quantified these components for cross-population comparisons [13, 34]. However, the SFI veers from these earlier works in its consideration and development of quantified and individualized health profiles. Rather than multiple intragroup and intergroup comparisons of single biomarkers (gross prevalences)—an analytical tool we advise using in conjunction with SFIs in the interpretation of biological health—skeletal frailty scores are calculated for each individual, thereby providing a distribution of overall frailty for the sample, which can be compared with other samples employing an identical SFI. This individualized, alternative approach to gross prevalence comparisons is a common practice in human biology and clinical research, and frailty scores and allostatic load so constructed have proven to be robust methods for interpreting and evaluating frailty patterns across age, gender, socioeconomic, and sociocultural groups [35–39].

The SFI was conceived with the intention of capturing four broad categories of somatic stress (Table 1): growth, infection/nutrition, activity, and trauma. Therefore, the 13 biomarkers comprising this index were selected as well-studied-and-confirmed barometers for these various health aspects. Biomarkers assessing growth stress included maximum femoral length (proxy for stature), maximum femoral head diameter (proxy for robusticity), and linear enamel hypoplasia (LEH) defects. Infection, nutritional deficiencies, and metabolic conditions often are difficult etiologies to tease out in reactive skeletal tissue. Therefore, skeletal biomarkers associated with any one or all three causes are grouped together into a broad infection/nutrition category: periosteal new bone (PNB) and osteomyelitis, periodontal disease (PD), porotic hyperostosis/cribra orbitalia (PH/CO), rickets/osteomalacia, osteoporosis, and neoplasms. Cumulative wear on the joints, a skeletal assessor of life-time activity, is gauged through osteoarthritis (OA), intervertebral disc disease (IVD), and rotator cuff disease (RCD). Healed and unhealed fractures characterize the final category, trauma.

**Table 1:** Skeletal biomarkers of stress incorporated into frailty indices (SFI) with designated scoring schemata, elements observed, and criteria for frailty. Outlined standards [52] were used to identify conditions of frailty: femoral head and maximum length [33]; linear enamel hypoplasia [71]; periosteal new bone [72] and osteomyelitis [14]; periodontal disease and osteoarthritis [73]; cribra orbitalia [74]; osteomalacia [75]; neoplastic disorders [14, 76]; osteoporosis and rotator cuff disease [77]; intervertebral disc disease [78]; and trauma [79].

Stress Category	Frailty Biomarkers	Scoring observations	Elements observed	Frailty Score “1”
Growth	Femoral Length Femoral Head Diameter Linear Enamel Hypoplasia	Lengths in quadrants Diameters in quadrants Presence/absence	Complete femora Complete femora All anterior dentition	Shortest lengths (¼)^a^^,^^b^ Smallest diameters (¼)^a^^,^^b^ Presence of single tooth with LEH ^c^
Nutrition and Infection	Periosteal New Bone (PNB)/ Osteomyelitis Periodontal Disease Cribra Orbitalia (CO) Osteomalacia Neoplasms Osteoporosis	Active, healing, absence Presence/absence Presence/absence Presence/absence Presence/absence Presence/absence	All preserved long bones Preserved mandibular and maxillary alveoli Preserved eye orbital plates Scapulae, vertebral column, ribs, sternum, and pelvic girdle All cranial and postcranial elements All long bones, ribs, and vertebrae	Active PNB or cloacae on long bone diaphysis or metaphysis surfaces Presence of >2mm alveolar resorption Presence of CO on right or left orbit Presence Presence Presence
Activity	Osteoarthritis (OA) Intervertebral Disc Disease (IVD) Rotator Cuff Disorder (RCD)	Presence/absence Presence/absence Presence/absence	All preserved joints (except uncovertebral, costovertebral, and costotransverse joints) All preserved vertebra Preserved humeri and scapulae	Presence of one joint with OA Presence of one vertebra with IVD Presence of RCD
Trauma	Fracture	Perimortem, antemortem, absence	All cranial and postcranial surfaces	Perimortem and antemortem trauma associated with soft tissue injuries or blunt force, sharp force, and projectile injuries

^a^Male femoral length quartiles: **x < 447mm**, 447mm ≤ x < 461mm, 461mm ≤ x < 475mm, x ≥ 475mm; female femoral length quartiles: **x < 414mm**, 414mm ≤ x< 434mm, 434mm ≤ x < 443mm, x ≥ 443mm (highest frailty measurements underlined)

^b^Male femoral head diameter quartiles: **x < 46.7mm**, 46.7mm ≤ x < 48.4mm, 48.4mm ≤ x < 49.9mm, x ≥ 49.9mm; female femoral head diameter quartiles: **x < 41.2mm**, 41.2mm ≤ x < 42.7mm, 42.7mm ≤ x < 45.4mm, x ≥ 45.4mm (highest frailty measurements underlined)

^c^The majority of individuals scored generally showed signs of two anterior teeth with at least one LEH lesion

The range of skeletal frailty scores depends upon the number of biomarkers included in the SFI. For the 13-biomarker SFI, individual skeletal frailty scores may range from “0” (low frailty) to “13” (high frailty). The scores are calculated by adding together values assigned to each biomarker; based on their risk level, relative to the overall sample, biomarkers are assigned “0” (low frailty) or “1” (high frailty) (Table 1). Most of the frailty variables—LEH, PD, PH/CO, neoplasms, OA, IVD, RCD, and fractures—are defined by presence or absence for high and low risk, respectively. For example, if an individual exhibits pathognomonic signs of OA, the researcher would mark a “1” for this biomarker. For the periosteal new bone (PNB) and osteomyelitis biomarker, three states (active, healing, and absent) are considered for scoring: active PNB suggests active infection or inflammatory response at the time of death of the individual, so this state is associated with high (“1”) frailty [40]. Since the etiology of PNB may be due to trauma, only active cranial and postcranial lesions, which are not specifically attributed to trauma in the database, are scored in the index, so as not to overrepresent trauma in the cumulative frailty score. Finally, the metric femoral variables require that the samples be divided into quartiles, smallest to largest measurements, for each biomarker. Individuals who have maximum femoral lengths or head diameter measurements falling into the lowest (smallest) quartile of the observed sample distribution are assigned a high risk “1” score for the specific biomarker. These definitions and explanations are further outlined in Table 1.

Although the SFI attempts to offer a readily applicable option for assessing skeletal “health” in past populations, Marklein and colleagues [1] acknowledged several shortcomings implicit in this binary approach. One of the first issues, which we address in this paper, is the limited number of skeletons in samples that meet the 13-biomarker criteria. In theory, and in an ideal world of primary, discrete burials and skeletal preservation, the SFI would include more than the original 13 biomarkers, and potentially subdivide current biomarkers into additional independent ones (e.g., cribra orbitalia and porotic hyperostosis). The authors maintain that more biomarkers, in addition to gross prevalence and mortality data, will provide a more complete rendering of factors contributing to frailty. Based on such a perspective, the 13-biomarker SFI is indeed more comprehensive than those with fewer biomarkers, but it is not likely to be the SFI “gold standard.” However, it was the maximum SFI that could be developed using this particular sample. Unfortunately, the more biomarkers included in the SFI, the smaller the sample size and, consequently, the sample’s representativeness of the whole population.

An additional concern with the proposed SFI method is the qualification of some biomarkers as evidence of physiological dysregulation or frailty. For example, some researchers have argued that LEH is evidence of resilience [27, 41]. As SFIs are applied exclusively to adults within a population, we suggest LEH presence is a vestige of childhood stress while absence indicates robust immunomodulatory capacities that counteracted juvenile stress or reduced exposure to childhood stress. Etiologies of biomarkers also require justification when incorporated in a site-specific SFI. Periosteal new bone (PNB), in particular, is commonly associated with non-specific infection in bioarchaeological studies [12], but this overlooks the responsiveness and activity of the periosteum [42]. For the SFI presented herein, we score active cranial and postcranial PNB, which are not explained by trauma in the WORD database, as high frailty. Unfortunately, preservation may hinder the ability to assess the etiology of lesions, so oftentimes bioarchaeologists may only have a left- or right-sided element to examine. Furthermore, even with relatively complete skeletons, PNB pathogenesis is difficult to identify [43]. Future applications of the SFI may choose to focus on a specific element (e.g., [25, 44]) for more refined etiological explanations of biomarkers like PNB.

A third, and more debatable, shortcoming of the SFI is its quantification of “high” and “low” risk evaluations of biomarkers as well as its equal weighting of biomarkers. Regarding the former, there may be variation in how a researcher differentiates between high and low risk based on the context of the sample. For example, in an archaeological sample where the majority of the population has died from perimortem trauma (e.g., Norris Farms [45]), it may by more informative to quantify an individual’s frailty before the massacre by assigning high risk (“1”) to an antemortem, healing fracture. Furthermore, in communities where there is endemic violence, a better representation of frailty differences may be to quantify interpersonal trauma as high risk (“1”) and accidental/unintentional trauma as low risk (“0”) [46]. Again, determinations of high and low risk for all biomarkers are context-specific and must be justified by the bioarchaeologists developing their own research- and population-specific SFIs. Equal weightings for all biomarkers is a crucial arena for future discussion, as evidenced by previous criticism of allostatic load and frailty indices in human biology [47]. There can be little argument that a malignant tumor, albeit equally weighted with active PNB, has a greater effect on overall somatic health. Nevertheless, if an individual died with active PNB, we cannot immediately rule out the causes of PNB contributing to the individual’s frailty at the time of death. For this reason, we do not proportionately weight lesions in the presented SFI. At the same time, we do not dismiss the possibility that proportionately weighted biomarkers based on clinical and epidemiological data may improve applications of SFIs to available samples.

### Expanding SFI applicability, maintaining explanatory power

As originally proposed and applied, the SFI comprised a suite of 13 metric and non-metric skeletal biomarkers of lifetime stress. In our original analysis of monastic and nonmonastic Medieval London cemetery samples, the 13-biomarker index criteria reduced the number of skeletons for which SFI could be determined (N = 134) to less than 12% of the original skeletons (N = 976), and only 23% of the sample (N = 558) examined earlier by DeWitte and colleagues [48]. This reduced sample size was a concern, as the 134 skeletons retained for analysis of skeletal frailty may not have been fully representative of the total population. A second concern was how useful the 13-biomarker SFI would be for more fragmented collections than the Medieval London ones used to develop it. In this paper, we address both of these concerns by constructing SFIs using exclusively nonmetric biomarkers (2–11). By excluding these two metric biomarkers, maximum femoral length and maximum femoral head diameter (proxies for stature and robusticity), the sample available for analyses increases, addressing our first concern. Subsequently, we compare reduced SFIs to results observed using the original 13-biomarker SFI. After reassessing differences by age and sex within and between Medieval London monastic and nonmonastic samples using the original sample of 134 and SFI constructs of 2 to 11 biomarkers, we apply a 6-biomarker nonmetric SFI to the same Medieval monastic and nonmonastic cemeteries, albeit with an increased sample size of 517 individuals. Last, we compare these results with those from a more parsimonious 4-biomarker nonmetric SFI, tailored to include one biomarker from each of four broad stressor categories, to determine if, for samples with poorer preservation, a simple SFI provides a similar picture of these individuals as the more comprehensive SFIs.

### Monastic and nonmonastic communities in Medieval London

The Medieval period in England and throughout Europe was characterized by religiously-and politically-enforced orders of social and economic status. Broadly, status groups included aristocratically-born elites, religious castes, and the landed rural and urban poor [49, 50]. While interactions between these groups were constant, there was little to no fluidity between social strata. Similarly, although individuals of all groups were exposed to comparable ecological and epidemiological climates, their abilities and resources to mitigate and buffer exposures were not equal [49, 51]. Analyzing human skeletal remains from six Medieval cemeteries, DeWitte and colleagues [48] demonstrated how these disparities between strata translated into differential mortality risks between, specifically, the monastic and nonmonastic communities in London. According to their findings, nonmonastic adults were at significantly higher risk of mortality than their monastic contemporaries. In this paper, we examine skeletal frailty between individuals from monastic and nonmonastic cemetery contexts, deriving our samples from the cemeteries utilized by DeWitte and colleagues [45], to assess whether monastic and nonmonastic lifeways had variable effects on the skeletons in these stratified groups.

## Materials

As detailed previously [1], all data used in these analyses are obtained from the Museum of London’s (MoL) open-access Wellcome Osteological Research Database (WORD). Therefore, all diagnoses of pathological lesions, sex estimations, and age assignments into one of four adult age categories are derived from data observed and published by specialists at the MoL’s Centre for Human Bioarchaeology (CHB), following standards outlined in the *Human Osteology Method Statement* [52] (see Table 1 for more details).

### Monastic samples

The representative monastic sample is a combination of individuals from Merton Priory (1117–1538 CE) and Bermondsey Abbey (1066–1540 CE) (Table 2). Merton Priory was the site of an Augustinian order of monks. As a center of learning in the Medieval period, the priory rose in wealth and prestige. Archaeological evidence from the Merton Priory cemetery—grave goods of lamps, chalices, and golden patens—attest to the richness affiliated with this monastic community [53, 54]. Individuals interred at Bermondsey Abbey are associated with the Cluniac order, a subset of the Benedictines [55]. This monastic community accrued wealth from its extensive estates throughout southern England [56].

**Table 2:** Chronological and demographic data for total monastic and nonmonastic samples from which this study’s smaller samples were derived.

	Cemetery	Dates	Total	Recovered individuals			
				Adult Males	Adult Females	Indeterminate Adults	Juveniles
Monastic	Bermondsey Abbey	1117–1538 CE	201	147 (73.1%)	0 (0.0%)	53 (26.4%)	1 (0.5%)
	Merton Priory	1066–1540 CE	676	485 (71.7%)	53 (7.8%)	105 (15.5%)	33 (4.8%)
	Total		877	632 (72.1%)	53 (6.0%)	158 (18.0%)	34 (3.9%)
Nonmonastic	Guildhall Yard	1140–1350 CE	68	18 (26.5%)	15 (22.1%)	14 (20.6%)	21 (30.9%)
	Spital Square	1200–1500 CE	124	43 (34.7%)	23 (18.5%)	16 (12.9%)	42 (33.9%
	St. Mary Graces	1350–1538 CE	389	136 (35%)	68 (17.5%)	79 (20.3%)	106 (27.2%)
	St. Benet Sherehog	1250–1666 CE	39	8 (20.5%)	4 (10.3%)	12 (30.8%)	15 (38.5%)
	Total		620	205 (33.1%)	110 (17.7%)	121(19.5%)	184 (29.7%)

### Nonmonastic samples

The representative nonmonastic, or lay, community in Medieval London comprises samples from Guildhall Yard (1140–1350 CE), Spital Square (1200–1500 CE), St. Mary Graces (1350–1538 CE), and St. Benet Sherehog (1250–1666 CE) (Table 2). Guildhall Yard was the burial site for lay members of the St. Lawrence Jewry parish. These individuals presumably lived in the neighboring tenement community [57]. Although associated with the Augustinian prior of St. Mary Spital, most of the individuals from the Spital Square cemetery were recovered from the hospital cemetery, into which were deposited the remains of the local urban poor [58]. The burial ground north of the Cistercian abbey of St. Mary Graces was delineated for the lay members of the parish, while the intramural inhumations were reserved for members of higher religious and economic status [59]. Finally, a fraction of the individuals exhumed from the St. Benet Sherehog cemetery, a smaller representative parish population, are included [60].

### 2- to 11-biomarker SFI nonmetric constructs

Originally, we used the 13-biomarker SFI to determine if frailty differentially affected individuals buried in Medieval London monastic and nonmonastic cemeteries [1]. The original sample included 134 individuals from six Medieval London sites. We use them to test comparability between the original 13-biomarker SFI and the 2–11 biomarker SFI constructs. These monastic and nonmonastic samples were based on the same cemeteries included in DeWitte and colleagues’ [48] earlier study investigating mortality differentials between monastic and lay communities: St. Merton’s Priory (N = 40) and Bermondsey Abbey (N = 34); Guildhall Yard (N = 12), Spital Square (N = 13), St. Mary Graces (N = 33), and St. Benet Sherehog (N = 2).

### Reduced multi-variable SFI constructs

When fewer biomarkers were incorporated into SFIs, the sample size increased from 134 to 517 individuals: St. Merton’s Priory (N = 303) and Bermondsey Abbey (N = 86); Guildhall Yard (N = 13), Spital Square (N = 20), St. Mary Graces (N = 90), and St. Benet Sherehog (N = 5). With this larger sample we estimated the nonmetric 6- and 4-biomarker SFIs. We then examined how SFI distributions by lifestyle (monastic or nonmonastic), age, and sex differed between these two reduced-biomarker SFI constructs and from the original 13-biomarker SFI.

## Methods

We designed and operationalized the SFI as a measure of individual skeletal frailty based upon conditions and traits likely to permanently mark the skeleton during life, making them biomarkers of lifetime frailty [1, 13, 34, 61]. Our initial estimate of skeletal frailty was a composite of 13 biomarkers (13-biomarker SFI), providing a theoretical range of SFI scores from 0 to 13, with zero representing lowest frailty and higher scores representing progressively greater frailty. All skeletal biomarkers were scored as “0” or “1” according to whether the condition qualified as being indicative of low or high frailty, respectively [1]. For most biomarkers, “1” was assigned to represent presence of a condition; for other biomarkers, scores of “0” or “1” were assigned according to the state of the observed pathological lesion, e.g., active versus healing/healed. Once each biomarker was assigned a score, zeros and ones were summed for each individual to represent that person’s specific SFI.

Based on these SFI criteria, frailty index scores were tabulated with 2–11 nonmetric biomarkers and the 13-metric-and-nonmetric SFI for the original, smaller sample (N = 134), and with the 6-biomarker SFI and parsimonious 4-biomarker SFI for the larger sample (N = 517) from Medieval London. Using nonparametric Spearman’s correlation coefficients in SPSS 24, we tested all SFI estimates (2-to-11 and 13-biomarker SFIs) for individuals in Medieval London monastic and nonmonastic samples to determine whether nonmetric SFIs significantly correlated with the original 13-biomarker SFI. A lack of significant correlation between the 13-biomarker SFI and any nonmetric SFI would suggest that the latter produces a different depiction of frailty, a statistically less informative alternative to the original 13-biomarker SFI. Contrarily, a significant Spearman’s correlation coefficient would suggest that nonmetric SFIs are capturing a similar frailty pattern within the sample, albeit with fewer recorded biomarkers.

We also performed Analysis of Variance/Covariance (ANOVA/ANCOVA) tests between age, sex, and burial context to see how means, standard deviations, explained variance, and significance (P-values) compared between 13-biomarker and nonmetric SFIs. We used ANCOVA when comparing SFIs between cemeteries and between sexes, assigning age as a covariate, because several of the biomarkers, notably irreversible degenerative joint conditions, correlate significantly with age. To analyze the relationship between age and SFI, we performed ANOVA tests with a post-hoc Tukey’s HSD test. From these results, we established which nonmetric SFIs comprising the fewest biomarkers maintained not only a significant correlation with the 13-biomarker SFI, while also yielding similar ANOVA/ANCOVA results, but also a correlation that deviated little from that of SFIs with more biomarkers.

For the 517 individual sample, we also conducted ANOVA and ANCOVA tests and compared results for the 6- and the more parsimonious 4-biomarker SFI distributions. The purpose of these comparisons was to determine whether fewer biomarkers/reduced-variable SFIs reflected results (e.g., explained variation, P-values) similar to the more comprehensive SFIs (i.e., ones with more biomarkers for frailty). All statistical analyses were conducted in SPSS 24, and the alpha value for significance was set as 0.05.

### Reducing variable contribution to SFI

Examination of Medieval London monastic and nonmonastic skeletal collections indicated that metric biomarkers, specifically maximum femoral length and head diameter biomarkers, most limited sample size. Therefore, we explored how well SFIs based upon fewer biomarkers correlated with/replicated the results of original 13-biomarker SFIs. Our method was to first drop biomarkers that most limited sample size from the total population, thereby increasing the sample size from 134 to 517. Following this, within the 134 originally included, we dropped those that were least frequently observed in both the total (517) and smaller sample (134), as they most limited sample size. Thereafter we removed biomarkers sequentially beginning with those least frequently observed among the combined monastic and nonmonastic Medieval London skeletal samples (N = 134) (Table 3). For example, only three cases of rickets/osteomalacia were reported, but 16 diagnoses of neoplasms were recorded in the 134 sample; therefore, the biomarker rickets/osteomalacia was removed third (after the two metrics) and a 10-biomarker index estimated. Following this, presence of neoplastic growth was removed and a 9-biomarker index estimated. Biomarkers were sequentially removed until only two (linear enamel hypoplasia and periodontal disease) remained. There is no need to examine single-biomarker SFIs, as they are identical to the biomarker’s gross prevalence in the sample. Among the available 11 nonmetric biomarkers, there are over 1,300 possible 2- to 11-biomarker SFI iterations. Our approach is pragmatic; we originally tested an index based upon all available biomarkers. Here we examined SFIs based upon the specific frailty biomarkers available for this Medieval population. Dropping biomarkers based upon their frequency allows us to first assess a maximum information SFI and then reduce this to an index that makes use of a more limited number of biomarkers, as may be available for another sample. In this particular sample pathological lesions observed at greatest frequency arguably represent conditions common in the London Medieval populations affecting most individuals, regardless of socioeconomic status, age, sex, or lifestyle. As an iterative process, our approach also reduces the likelihood of observing a significant difference in SFI by sex, age, or social status, increasing its methodological rigor.

**Table 3:** Non-metric multi-biomarker skeletal frailty index (SFI) constructs.

	Non-metric Skeletal Biomarkers of Frailty
11-variable SFI	PD, LEH, IVD, PNB, Fracture, OA, PH/CO, Neoplasm, RCD, Rickets/Osteomalacia, Osteoporosis^a^
10-variable SFI	PD, LEH, IVD, PNB, Fracture, OA, PH/CO, Neoplasm, RCD, Rickets/Osteomalacia
9-variable SFI	PD, LEH, IVD, PNB, Fracture, OA, PH/CO, Neoplasm, RCD
8-variable SFI	PD, LEH, IVD, PNB, Fracture, OA, PH/CO, Neoplasm
7-variable SFI	PD, LEH, IVD, PNB, Fracture, OA, PH/CO
6-variable SFI	PD, LEH, IVD, PNB, Fracture, OA
5-variable SFI	PD, LEH, IVD, PNB, Fracture
4-variable SFI	PD, LEH, IVD, PNB
3-variable SFI	PD, LEH, IVD
2-variable SFI	PD, LEH

^a^Periodontal disease (PD), linear enamel hypoplasia (LEH), intervertebral disc disease (IVD), periosteal new [active] bone (PNB), osteoarthritis (OA), porotic hyperostosis/cribra orbitalia (PH/CO), and rotator cuff disorder (RCD)

### Constructing a 4-biomarker SFI

To construct a SFI useful across a broad range of human osteological assemblages, we examined SFIs wherein the number of contributing biomarkers ranged from 2 to 11 variables and also determined a specific 4-biomarker SFI that might be useful across multiple samples. For the 4-variable SFI, each biomarker was chosen to represent one category of stressors: growth (linear enamel hypoplasia), infection/nutrition (periosteal lesions), activity (osteoarthritis), and trauma (fracture). Aside from osteoarthritis and periosteal lesions, LEH and trauma were commonly observed pathological lesions within both the original monastic and nonmonastic samples (N = 134). Intervertebral disc disease (IVD) and periodontal disease (PD) also were observed at high frequencies in these Medieval London samples (IVD: 343/517, 66%; PD: 387/517, 75%). We suggest that IVD and PD likely indicate a general baseline of frailty across the total population. Most individuals have IVD and/or PD, suggesting these conditions were common in Medieval London. Our suggestion is that these biomarkers reflect similarities in exposures between samples and may little differentiate frailty indices; consequently, IVD and PD have not been included in the 4-biomarker SFI. Similarly, periosteal lesions were selected as the biomarker of infection, rather than PD. The latter is more strongly correlated with age, while periosteal lesions are reversible and do not always show age biases in their etiologies [62, 63; (contra [64]}]. LEH were observed at relatively high frequency (53%) among both monastic and nonmonastic samples. However, as the sole nonmetric biomarker of growth remaining, after removing femoral length and robusticity, LEH was retained in the 4-biomarker SFI.

## Results

### Medieval monastic and nonmonastic samples

**Fig 1:**
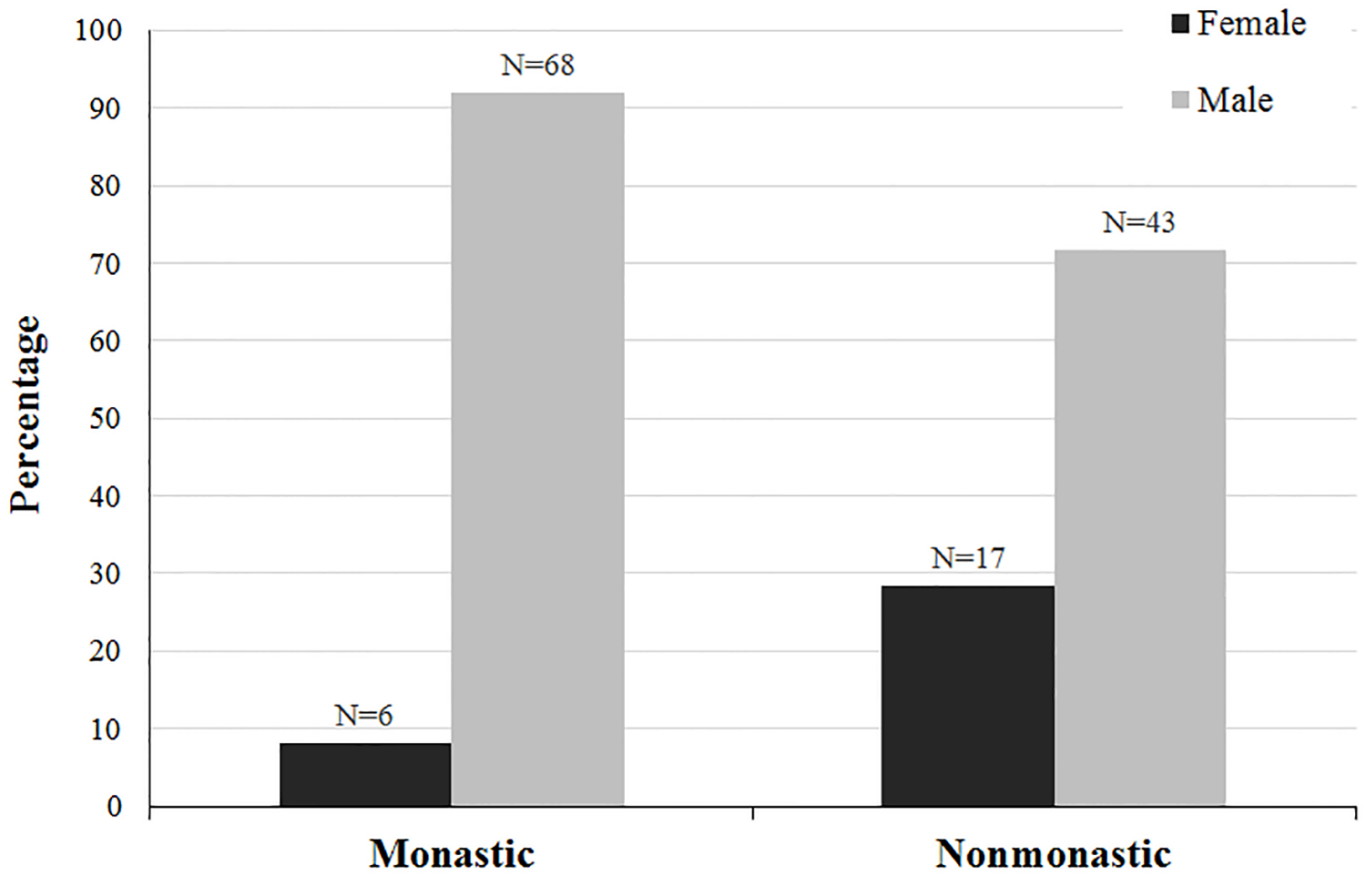
Distribution of 134 adult males and females from Medieval London monastic and nonmonastic cemetery contexts.

**Fig 2:**
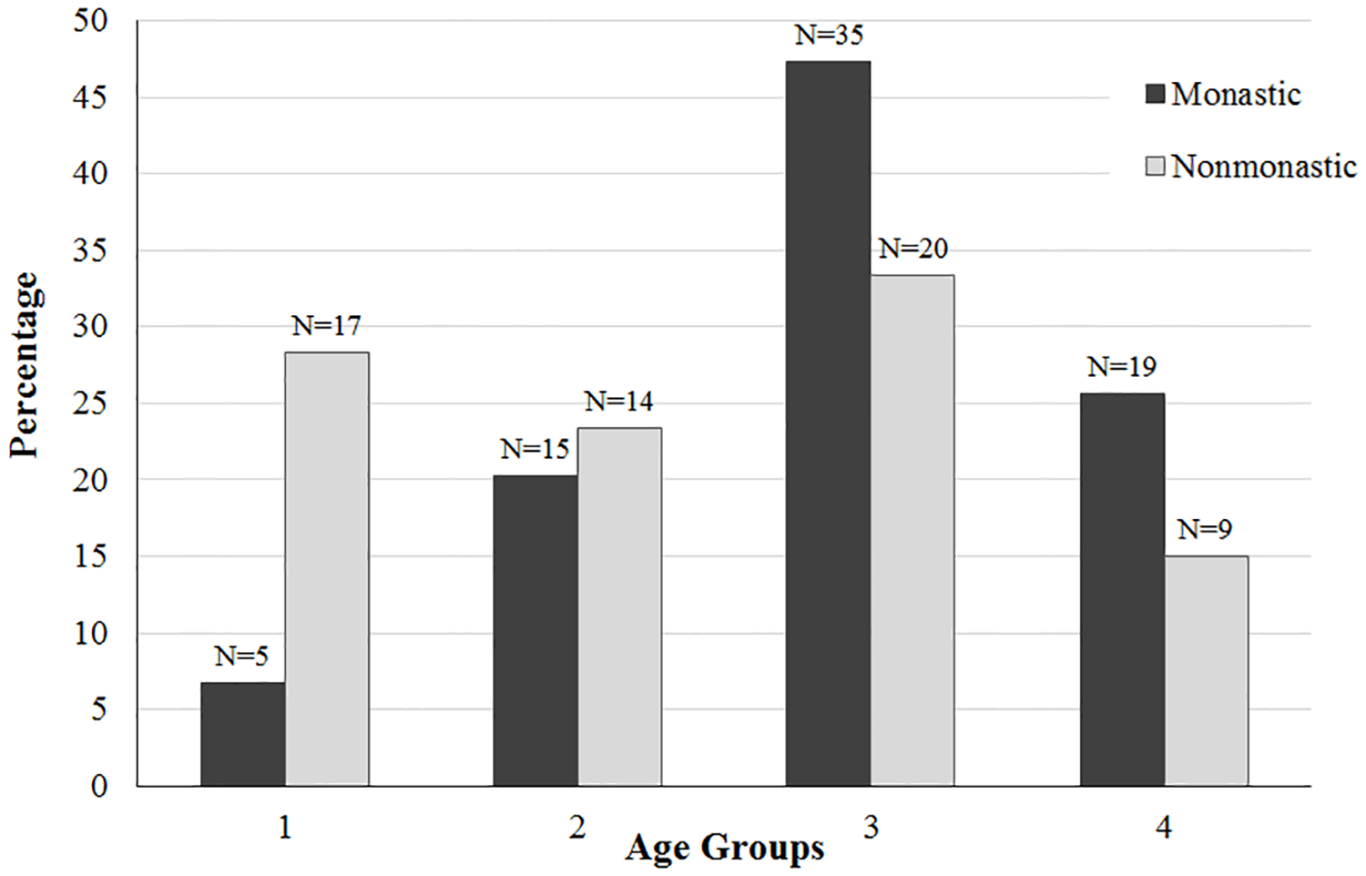
Age distribution of 134 adults from Medieval London monastic and nonmonastic cemetery contexts.

**Fig 3:**
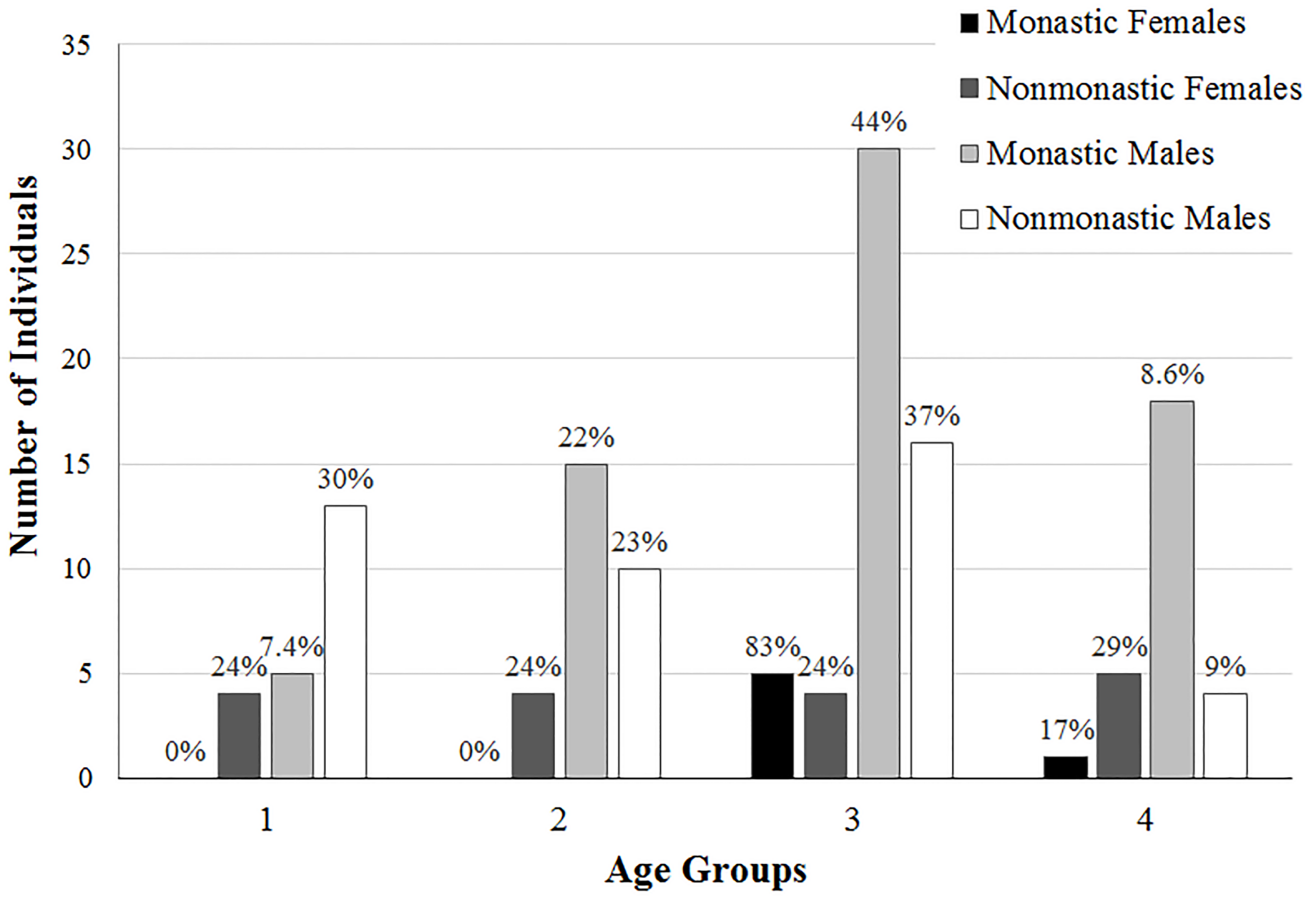
Distribution by age and sex of 134 adult males and females from Medieval London monastic and nonmonastic cemetery contexts. Percentages of subgroups represented by each age category.

**Fig 4:**
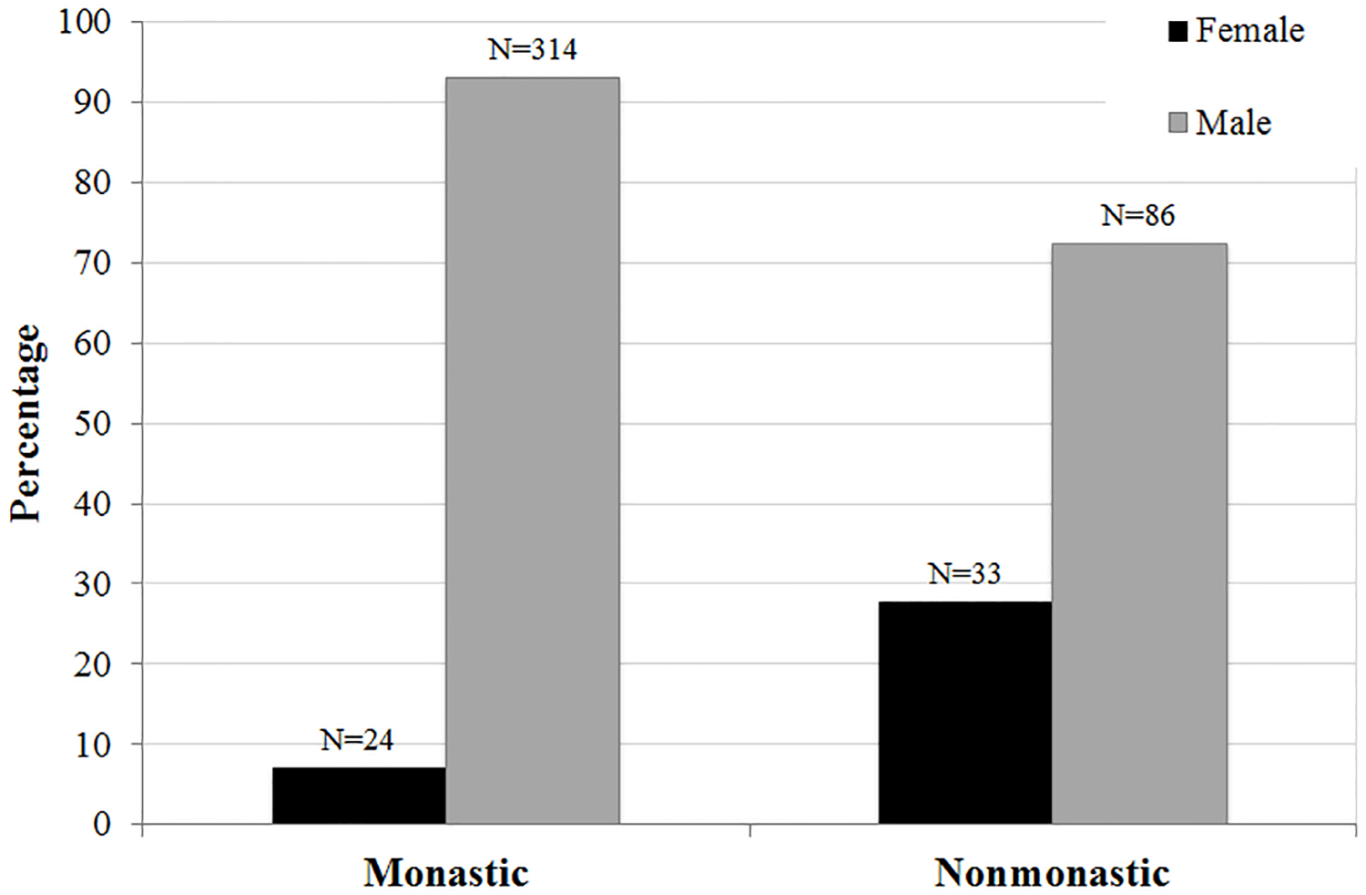
Distribution of 517 adult males and females from Medieval London monastic and nonmonastic cemetery contexts.

**Fig 5:**
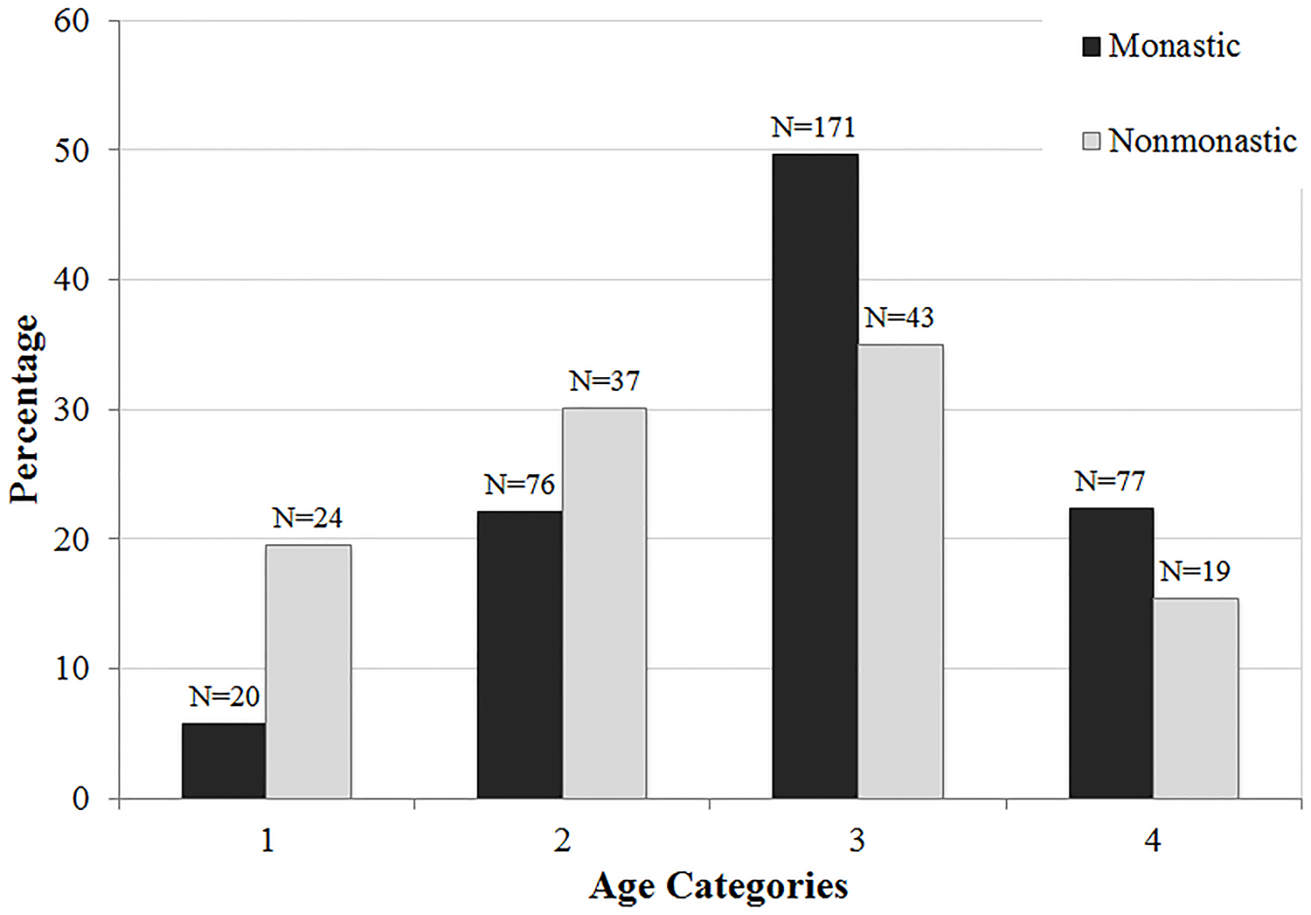
Age distribution of 517 adults from Medieval London monastic and nonmonastic cemetery contexts.

**Fig 6:**
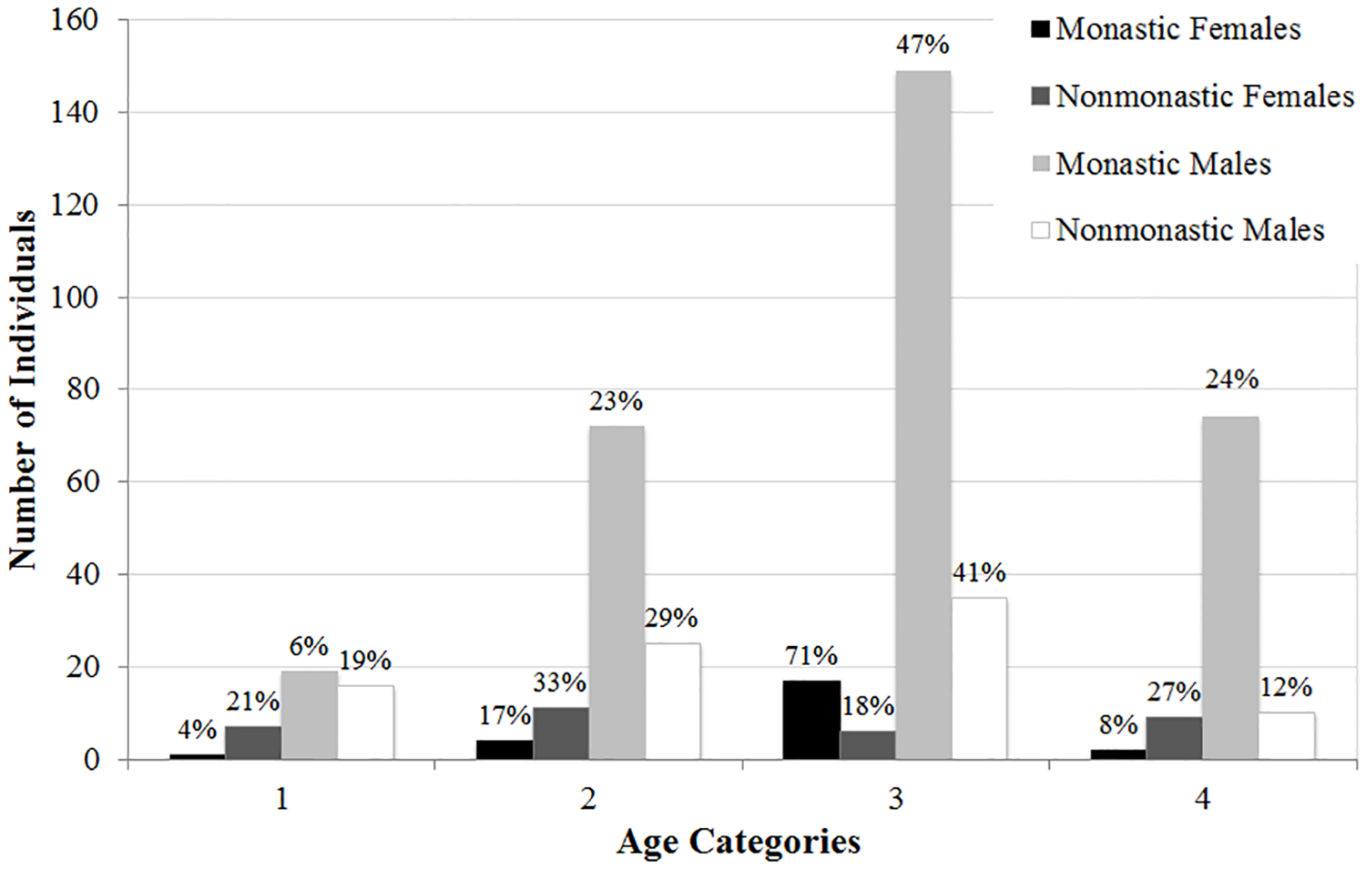
Distribution by age and sex of 517 adult males and females from Medieval London monastic and nonmonastic cemetery contexts. Percentages of subgroups represented by each age category.

The demographic breakdowns of Medieval monastic and nonmonastic samples (N = 134 and N = 517) are shown in Tables 4–9 and Figs 1–6. For the 134 individual sample, data indicate a disproportionate representation of males: monastic sample, 91.9% adult male; nonmonastic sample, 71.7% adult male (Table 4, Fig 1). This pattern holds, almost identically for the larger individual sample (N = 517): 92.9% of monastic and 72.2% of nonmonastic samples are males (Table 7, Fig 4). For both the reduced (N = 134) and larger (N = 517) samples, age distributions also are uneven. In the 134 skeletons, the monastic sample is dominated by two older age groups (36 to 45 and over 45 years; 73%); in the nonmonastic sample, these older age groups represent only 48% (Tables 5 and 8, Figs 2 and 5). Examining sex by age distribution in the 134 individuals, it becomes apparent in both settings how samples are skewed in favor of older males in monastic cemeteries (>65%) and younger males in nonmonastic cemeteries (>38%) (Table 6, Fig 3). For the larger sample (N = 517), older males (≥36 years) dominate the monastic sample (>65%), and the oldest (≥45 years) and youngest (18–25 years) females represent less than a percent of the monastic sample. Middle aged males (26–45 years) comprise over 50% of the nonmonastic sample, with only 30% comprised of females (Table 9, Fig 6).

**Table 4:** Number of adult males and females from the total sample of 1497 Medieval monastic and nonmonastic individuals, with all 13 biomarkers available (N = 134) [1].

	N	Females	Males
Monastic	74	6 (8.1%)	68 (91.9%)
Nonmonastic	60	17 (28.3%)	43 (71.7%)

**Table 5:** Age distribution of 134 skeletons from Medieval monastic and nonmonastic cemeteries [1].

Age category		N	Monastic	Nonmonastic
1	18–25 years	22	5 (6.76%)	17 (28.33%)
2	26–35 years	29	15 (20.27%)	14 (23.33%)
3	36–45 years	55	35 (47.30%)	20 (33.33%)
4	>45 years	28	19 (25.68%)	9 (15.00%)

**Table 6:** Distribution of adult males and females according to age categories in original Medieval monastic and nonmonastic cemeteries. Percentages reflect proportion of monastic or nonmonastic sample.

Age category		Monastic (N = 74)		Nonmonastic (N = 60)	
		Females	Males	Females	Males
1	18–25 years	0 (0%)	5 (6.76%)	4 (6.67%)	13 (21.67%)
2	26–35 years	0 (0%)	15 (20.37%)	4 (6.67%)	10 (16.67%)
3	36–45 years	5 (6.76%	30 (40.54%)	4 (6.67%)	16 (26.67%)
4	>45 years	1 (1.35%)	18 (24.32%)	5 (8.33%)	4 (6.67%)

**Table 7:** Number of adult males and females from 1497 skeletons of Medieval monastic and nonmonastic cemeteries with all 11 nonmetric biomarkers available (N = 517).

	N	Females	Males
Monastic	338	24 (7.10%)	314 (92.9%)
Nonmonastic	119	33 (27.7%)	86 (72.3%)

**Table 8:** Age distribution of from 1497 skeletons of Medieval monastic and nonmonastic cemeteries with all 11 nonmetric biomarkers available (N = 517).

Age category		N	Monastic	Nonmonastic
1	18–25 years	44	20 (5.81%)	24 (19.5%)
2	26–35 years	113	76 (22.1%)	37 (30.1%)
3	36–45 years	214	171 (49.7%)	43 (35.05%)
4	>45 years	96	77 (22.4%)	19 (15.4%)

**Table 9:** Distribution of adult males and females according to age categories in from increased sample of Medieval monastic and nonmonastic cemeteries (N = 517). Percentages reflect proportion of monastic or nonmonastic sample.

Age category		Monastic (N = 338)		Nonmonastic (N = 119)	
		Females	Males	Females	Males
1	18–25 years	1 (0.30%)	19 (5.62%)	7 (5.88%)	16 (13.4%)
2	26–35 years	4 (1.18%)	72 (21.3%)	11 (9.24%)	25 (21.0%)
3	36–45 years	17 (5.03%)	149 (44.1%)	6 (5.04%)	35 (29.4%)
4	>45 years	2 (0.59%)	74 (21.9%)	9 (7.56%)	10 (8.40%)

### Frailty in Medieval monastic and nonmonastic samples

Results using Spearman’s correlation coefficients and ANOVA/ANCOVA are shown in Tables 10–18. Spearman’s correlation coefficients between the 13-biomarker SFIs and the 2- to 11-biomarker SFI constructs indicate significant correlations (0.645 to 0.861) with nonmetric SFIs (Table 10; row 1). A perceptible drop (~4%) in these correlations occurs when reducing the index from six (R = 0.852, P-value<0.005) to five biomarkers (R = 0.818, P-value<0.005) and is almost seven times as large a decline as any earlier decline. Overall, the lowest correlations are observed between the 13-biomarker and 2-biomarker SFI values, while correlation coefficients among the 4- to 11-biomarker SFIs all are greater than 0.800, illustrating a general similarity across SFIs using multiple biomarker constructs. However, none of the 2–11 biomarker SFIs achieves greater than a 0.860 correlation (R^2^ = 0.74) with the original 13-biomarker SFI, which remains our current standard index for estimating skeletal frailty.

**Table 10:** Spearman’s correlations for 2–11 and 13-biomarker skeletal frailty indices (SFIs) (R = correlation coefficient, P = P-value, and N = number of cases).

		13-SFI	11-SFI	10-SFI	9-SFI	8-SFI	7-SFI	6-SFI	5-SFI	4-SFI	3-SFI	2-SFI
13-SFI	R	1.00	.861	.858	.858	.857	.857	.852	.818	.820	.766	.645
	P		.000	.000	.000	.000	.000	.000	.000	.000	.000	.000
	N	134	134	134	134	134	134	134	134	134	134	134
11-SFI	R	.861	1.00	.998	.998	.998	.992	.978	.913	.908	.873	.655
	P	.000		.000	.000	.000	.000	.000	.000	.000	.000	.000
	N	134	134	134	134	134	134	134	134	134	134	134
10-SFI	R	.858	.998	1.00	1.00	1.00	.994	.980	.912	.907	.870	.654
	P	.000	.000			.000	.000	.000	.000	.000	.000	.000
	N	134	134	134	134	134	134	134	134	134	134	134
9-SFI	R	.858	.998	1.000	1.00	1.00	.994	.980	.912	.907	.870	.654
	P	.000	.000			.000	.000	.000	.000	.000	.000	.000
	N	134	134	134	134	134	134	134	134	134	134	134
8-SFI	R	.857	.998	1.000	1.00	1.00	.994	.980	.913	.908	.870	.654
	P	.000	.000	.000	.000		.000	.000	.000	.000	.000	.000
	N	134	134	134	134	134	134	134	134	134	134	134
7-SFI	R	.857	.992	.994	.994	.994	1.00	.993	.929	.924	.885	.672
	P	.000	.000	.000	.000	.000		.000	.000	.000	.000	.000
	N	134	134	134	134	134	134	134	134	134	134	134
6-SFI	R	.852	.978	.980	.980	.980	.993	1.00	.935	.929	.890	.672
	P	.000	.000	.000	.000	.000	.000		.000	.000	.000	.000
	N	134	134	134	134	134	134	134	134	134	134	134
5-SFI	R	.818	.913	.912	.912	.913	.929	.935	1.00	.988	.933	.716
	P	.000	.000	.000	.000	.000	.000	.000		.000	.000	.000
	N	134	134	134	134	134	134	134	134	134	134	134
4-SFI	R	.820	.908	.907	.907	.908	.924	.929	.988	1.00	.948	.741
	P	.000	.000	.000	.000	.000	.000	.000	.000		.000	.000
	N	134	134	134	134	134	134	134	134	134	134	134
3-SFI	R	.766	.873	.870	.870	.870	.885	.890	.933	.948	1.00	.804
	P	.000	.000	.000	.000	.000	.000	.000	.000	.000		.000
	N	134	134	134	134	134	134	134	134	134	134	134
2-SFI	R	.645	.655	.654	.654	.654	.672	.672	.716	.741	.804	1.00
	P	.000	.000	.000	.000	.000	.000	.000	.000	.000	.000	
	N	134	134	134	134	134	134	134	134	134	134	134

**Table 11:** ANCOVA results for multi-variable skeletal frailty indices (SFIs) comparing frailty between Medieval monastic (Bermondsey Abbey and Merton Priory) and nonmonastic (Guildhall Yard, St. Mary Graces, Spital Square, and St. Benet Sherehog) samples (N = 134).

	Monastic (N = 74)	Nonmonastic (N = 60)	Sig. (age)	Sig. (lifestyle)	Sig. (model)	R^2^
13-biomarker SFI	3.42 (1.4)	2.80 (1.5)	0.102	0.058	0.013	0.049
11-biomarker SFI	2.74 (1.2)	2.02 (1.1)	0.012	0.005	0.000	0.121
10-biomarker SFI	2.74 (1.2)	2.00 (1.1)	0.008	0.004	0.000	0.131
9-biomarker SFI	2.74 (1.2)	2.00 (1.1)	0.008	0.004	0.000	0.131
8-biomarker SFI	2.73 (1.2)	2.00 (1.1)	0.010	0.004	0.000	0.127
7-biomarker SFI	2.70 (1.2)	2.00 (1.1)	0.008	0.006	0.000	0.125
6-biomarker SFI	2.69 (1.2)	1.98 (1.1)	0.003	0.008	0.000	0.135
5-biomarker SFI	2.34 (0.98)	1.85 (1.0)	0.079	0.030	0.005	0.064
4-biomarker SFI	2.31 (0.94)	1.80 (0.99)	0.035	0.020	0.001	0.084
3-biomarker SFI	2.19 (0.84)	1.62 (0.80)	0.018	0.002	0.000	0.133
2-biomarker SFI	1.61 (0.62)	1.35 (0.63)	0.630	0.034	0.056	0.028

**Table 12:** ANOVA results for multi-variable skeletal frailty indices (SFIs) comparing frailty between age categories in Medieval monastic (Bermondsey Abbey and Merton Priory) and nonmonastic (Guildhall Yard, St. Mary Graces, Spital Square, and St. Benet Sherehog) samples (N = 134).

	18–25 years (N = 22)	26–35 years (N = 29)	36–45 years (N = 55)	Over 45 years (N = 28)	Sig. (age)	R^2^
13-biomarker SFI	3.00 (1.3)	2.62 (1.5)	3.18 (1.4)	3.71 (1.5)	0.041	0.04
11-biomarker SFI	2.09 (1.1)	1.90 (0.98)	2.55 (1.23)	2.96 (1.2)	0.003	0.082
10-biomarker SFI	2.05 (1.0)	1.90 (0.98)	2.55 (1.23)	2.96 (1.2)	0.002	0.086
9-biomarker SFI	2.05 (1.0)	1.90 (0.98)	2.55 (1.23)	2.96 (1.2)	0.002	0.086
8-biomarker SFI	2.05 (1.0)	1.90 (0.98)	2.55 (1.23)	2.93 (1.2)	0.003	0.083
7-biomarker SFI	2.00 (0.97)	1.90 (0.98)	2.55 (1.23)	2.89 (1.1)	0.003	0.083
6-biomarker SFI	1.91 (0.97)	1.90 (0.98)	2.55 (1.23)	2.89 (1.1)	0.001	0.091
5-biomarker SFI	1.86 (0.94)	1.83 (0.89)	2.22 (1.1)	2.43 (0.96)	0.078	0.029
4-biomarker SFI	1.73 (0.83)	1.83 (0.89)	2.20 (1.1)	2.39 (0.92)	0.038	0.041
3-biomarker SFI	1.55 (0.74)	1.72 (0.75)	2.04 (0.90)	2.25 (0.89)	0.013	0.058
2-biomarker SFI	1.32 (0.72)	1.52 (0.57)	1.53 (0.63)	1.54 (0.64)	0.577	-0.008

**Table 13:** ANCOVA results for multi-variable skeletal frailty indices (SFIs) comparing frailty between sexes in Medieval monastic (Bermondsey Abbey and Merton Priory) and nonmonastic (Guildhall Yard, St. Mary Graces, Spital Square, and St. Benet Sherehog) samples (N = 134).

	Males (N = 111)	Females (N = 23)	Sig. (age)	Sig. (sex)	Sig. (model)	R^2^
13-biomarker SFI	3.20 (1.5)	2.87 (1.5)	0.023	0.029	0.046	0.031
11-biomarker SFI	2.46 (1.2)	2.22 (1.2)	0.001	0.302	0.002	0.075
10-biomarker SFI	2.45 (1.2)	2.22 (1.2)	0.000	0.311	0.001	0.082
9-biomarker SFI	2.45 (1.2)	2.22 (1.2)	0.000	0.311	0.001	0.082
8-biomarker SFI	2.44 (1.2)	2.22 (1.2)	0.001	0.325	0.002	0.078
7-biomarker SFI	2.42 (1.2)	2.22 (1.2)	0.000	0.356	0.002	0.080
6-biomarker SFI	2.41 (1.2)	2.22 (1.2)	0.000	0.388	0.001	0.091
5-biomarker SFI	2.15 (1.0)	1.96 (0.98)	0.014	0.350	0.033	0.036
4-biomarker SFI	2.11 (0.99)	1.96 (0.98)	0.004	0.433	0.013	0.050
3-biomarker SFI	1.97 (0.87)	1.74 (0.86)	0.001	0.183	0.002	0.077
2-biomarker SFI	1.51 (0.63)	1.39 (0.66)	0.248	0.380	0.361	0.000

**Table 14:** ANCOVA results for multi-variable skeletal frailty indices (SFIs) comparing frailty between males and between females in Medieval monastic (Bermondsey Abbey and Merton Priory) and nonmonastic (Guildhall Yard, St. Mary Graces, Spital Square, and St. Benet Sherehog) samples (N = 134).

	Monastic Males (N = 68)	Nonmonastic Males (N = 43)	Sig. (age)	Sig. (lifestyle)	Sig. (model)	R^2^
13-biomarker SFI	3.12 (0.16)	2.77 (0.23)	0.116	0.474	0.128	0.020
11-biomarker SFI	2.71 (1.2)	2.07 (1.2)	0.026	0.051	0.002	0.092
10-biomarker SFI	2.71 (1.2)	2.05 (1.2)	0.018	0.042	0.001	0.103
9-biomarker SFI	2.71 (1.2)	2.05 (1.2)	0.018	0.042	0.001	0.103
8-biomarker SFI	2.69 (1.1)	2.05 (1.2)	0.022	0.043	0.001	0.099
7-biomarker SFI	2.66 (1.1)	2.05 (1.2)	0.017	0.059	0.001	0.097
6-biomarker SFI	2.65 (1.1)	2.02 (1.1)	0.008	0.066	0.001	0.110
5-biomarker SFI	2.31 (0.98)	1.91 (1.1)	0.104	0.165	0.037	0.042
4-biomarker SFI	2.28 (0.93)	1.84 (1.0)	0.047	0.114	0.010	0.065
3-biomarker SFI	2.16 (0.84)	1.67 (0.84)	0.021	0.035	0.001	0.104
2-biomarker SFI	1.59 (0.63)	1.40 (0.62)	0.651	0.181	0.265	0.006
	Monastic Females (N = 6)	Nonmonastic Females (N = 17)	Sig. (age)	Sig. (lifestyle)	Sig. (model)	R^2^
13-biomarker SFI	3.83 (0.48)	2.41 (0.29)	0.725	0.030	0.063	0.165
11-biomarker SFI	3.17 (1.5)	1.88 (0.86)	0.235	0.035	0.029	0.227
10-biomarker SFI	3.17 (1.5)	1.88 (0.86)	0.235	0.035	0.029	0.227
9-biomarker SFI	3.17 (1.5)	1.88 (0.86)	0.235	0.035	0.029	0.227
8-biomarker SFI	3.17 (1.5)	1.88 (0.86)	0.235	0.035	0.029	0.227
7-biomarker SFI	3.17 (1.5)	1.88 (0.86)	0.235	0.035	0.029	0.227
6-biomarker SFI	3.17 (1.5)	1.88 (0.86)	0.235	0.035	0.029	0.227
5-biomarker SFI	2.67 (1.0)	1.71 (0.85)	0.459	0.092	0.086	0.139
4-biomarker SFI	2.67 (1.0)	1.71 (0.85)	0.459	0.062	0.086	0.139
3-biomarker SFI	2.50 (0.84)	1.47 (0.72)	0.449	0.017	0.026	0.237
2-biomarker SFI	1.83 (0.41)	1.24 (0.66)	0.789	0.075	0.154	0.087

**Table 15:** ANCOVA results for 6-variable and 4-variable skeletal frailty indices (SFIs) comparing frailty between Medieval monastic (Bermondsey Abbey and Merton Priory) and nonmonastic (Guildhall Yard, St. Mary Graces, Spital Square, and St. Benet Sherehog) samples (N = 517).

	Mean (Stdev)	N	Sig. (age)	Sig. (lifestyle)	Sig. (model)	R^2^
**6-biomarker SFI**						
Monastic	2.69 (1.2)	338	0.000	0.000	0.000	0.084
Nonmonastic	2.12 (1.1)	119				
**4-biomarker SFI**						
Monastic	1.32 (0.94)	338	0.000	0.17	0.000	0.037
Nonmonastic	1.11 (0.84)	119				

**Table 16:** ANOVA results for multi-variable skeletal frailty indices (SFIs) comparing frailty between age categories in Medieval monastic (Bermondsey Abbey and Merton Priory) and nonmonastic (Guildhall Yard, St. Mary Graces, Spital Square, and St. Benet Sherehog) samples (N = 517).

	Mean (Stdev)	N	Sig. (age)	R^2^
**6-biomarker SFI**				
18–25 years	2.02 (1.05)	44	0.000	0.052
26–35 years	2.27 (1.04)	113		
36–45 years	2.72 (1.12)	214		
Over 45 years	2.89 (1.40)	96		
**4-biomarker SFI**				
18–25 years	1.07 (0.66)	44	0.000	0.036
26–35 years	1.04 (0.74)	113		
36–45 years	1.35 (0.92)	214		
Over 45 years	1.54 (1.1)	96		

**Table 17:** ANCOVA results for multi-variable skeletal frailty indices (SFIs) comparing frailty between sexes in Medieval monastic (Bermondsey Abbey and Merton Priory) and nonmonastic (Guildhall Yard, St. Mary Graces, Spital Square, and St. Benet Sherehog) samples (N = 517).

	Mean (Stdev)	N	Sig. (age)	Sig. (sex)	Sig. (model)	R^2^
**6-biomarker SFI**						
Male	2.57 (0.06)	400	0.000	0.773	0.000	0.050
Female	2.43 (0.15)	57				
**4-biomarker SFI**						
Male	1.26 (0.92)	400	0.000	0.647	0.000	0.033
Female	1.32 (0.89)	57				

**Table 18:** ANCOVA results for 6-variable and 4-variable skeletal frailty indices (SFIs) comparing frailty between females and between males in Medieval monastic (Bermondsey Abbey and Merton Priory) and nonmonastic (Guildhall Yard, St. Mary Graces, Spital Square, and St. Benet Sherehog) samples (N = 517).

	Mean (Stdev)	N	Sig. (age)	Sig. (cemetery)	Sig. (model)	R^2^
**6-biomarker SFI**						
Monastic females	2.80 (0.26)	24	0.058	0.103	0.026	0.094
Nonmonastic females	2.17 (0.18)	33				
**4-biomarker SFI**						
Monastic females	1.40 (0.20)	24	0.021	0.931	0.061	0.065
Nonmonastic females	1.26 (0.14)	33				
	Mean (Stdev)	N	Sig. (age)	Sig. (cemetery)	Sig. (model)	R^2^
6-biomarker SFI						
Monastic males	2.68 (0.063)	314	0.000	0.001	0.000	0.073
Nonmonastic males	2.12 (0.12)	86				
4-biomarker SFI						
Monastic males	1.32 (0.049)	314	0.002	0.074	0.000	0.034
Nonmonastic males	1.04 (0.090)	86				

After estimating skeletal frailty in Medieval London monastic and nonmonastic samples with SFIs constructed using different aggregations of frailty biomarkers, we tested these SFIs for significant associations with sex, age, and lifestyle using ANOVA and ANCOVA (Tables 11–14). As previously reported, the 13-biomarker SFI differs significantly between monastic and non-monastic burials (Table 11), likely reflecting differences in lifestyles between residents in these two settings during life [1]. Using the nonmetric SFI constructs, individuals buried in monastic cemeteries show significantly higher (P<0.05) average frailty for all but the 2-biomarker SFI. With age included as a covariate, over 12% of total observed variation in the 6- through 13-biomarker SFIs is explained by having lived a monastic or nonmonastic lifestyle (Table 11). When only five or fewer skeletal frailty biomarkers are included, statistical significance of age is reduced and explained variance drops to 6%, but explained variance rebounds to 13.3% when only 3 biomarkers are included (Table 11).

Age is associated significantly with all SFI biomarker composites examined, with frailty generally increasing from the youngest to the oldest categories (Table 12; S1 Table). Nevertheless, observed patterns of variation across age categories differ between SFI constructs. For the 13-biomarker SFI, the youngest category (ages 18 to 25 years) exhibits higher average SFI than the immediate following age category (ages 26 to 35 years). This pattern of higher SFIs at the youngest ages is observed for all SFI constructs including five or more biomarkers, but not those with four or fewer biomarkers. With 2–5 biomarker SFIs, average frailty scores increase monotonically from the youngest to oldest age categories, failing to reveal early life stress experiences clearly illustrated by the 13-biomarker index.

Apart from the 2-biomarker model, all nonmetric SFIs show significant differences by sex (Table 13). Examination of ANCOVA results indicates age is a major contributor to SFI differences by sex. Although both age and sex significantly influence the 13-biomarker SFI, they explain less than 4% of its total variation. Examining males and females separately and comparing between monastic and nonmonastic cemetery groups, SFIs are significantly higher among monastic skeletons than nonmonastic samples for all nonmetric SFIs, sans the 2-biomarker SFIs (Table 14). Although age significantly influences the SFI differences between monastic and nonmonastic males (Table 14), age shows no significant association with SFI between monastic and nonmonastic females. However, lifestyle explains significant SFI variation (16.5%-23.7%) between female subgroups (Table 14). This occurs despite the fact that all monastic females lived an estimated 36 years or more, while only half (9 of 17, 53%) of nonmonastic females attained these older ages (Table 6, Fig 3).

### Comparing 6- and 4-biomarker SFI

To determine the effect of fewer biomarkers on associations of SFIs with age, sex, and lifestyle, we compared results using a 4- and 6-biomarker index to those with our original 13-biomarker SFI. We selected the 6-biomarker from the 2- to 11-biomarker SFIs, as it was the fewest biomarker index that remained as highly correlated with the original 13-biomarker SFI and reproduced similar associations with age, sex, and lifestyle. We estimated a more parsimonious 4-biomarker SFI to include one representative component from each of the four “health” categories. By reducing the number of included biomarkers, the available sample size from monastic and nonmonastic cemeteries increased to 517. Using ANOVA and ANCOVA, significant differences in these two SFIs by age, sex, and lifestyle are observed (Tables 15–18). For both the 4- and 6-biomarker SFIs, monastic skeletons exhibit higher frailty (1.32, 2.69) than nonmonastic skeletons (1.11, 2.12). However, age is a more significant contributor to the observed 4-biomarker SFI difference. Both age and lifestyle are significant predictors of the difference in 6-biomarker SFIs between monastic and nonmonastic samples. Based upon ANCOVA regression models, lifestyle explains 8.4% of variation in 6-biomarker SFI, but only 3.7% in the 4-biomarker SFI.

With age as an independent variable, SFIs differ significantly across age categories (Table 16; S2 Table). Age explains 3.6% and 5.2% of variation in the 4- and 6-biomarker SFIs, respectively. Using 6 biomarkers, average skeletal frailty increases monotonically from the youngest (18–25 years) to oldest (≥45 years) age group. Conversely, the 4-biomarker SFI decreases from the first (18–25 years) to second (26–35 years) age category, as does the 13-biomarker SFI, although this difference is not significant for the 4-biomarker index.

Both intersexual and intrasexual differences in SFI distributions are observed for the 4- and 6-biomarker indices (Tables 17 and 18). The 6-biomarker SFI differs significantly between males (SFI = 2.57) and females (SFI = 2.40). However, in the regression model, sex does not contribute significantly to skeletal frailty, but age does (Table 17). For the 4-biomarker SFI, male (SFI = 1.26) and female (1.32) averages also differ, but in the opposite direction, and this discrepancy is influenced mainly by age (P-value<0.005), not sex (P-value = 0.647). Monastic males exhibit higher average skeletal frailty than nonmonastic males (Table 17). For the 6-biomarker SFI, only 7.3% of its total variation is explained by lifestyle (Table 18). For the 4-biomarker SFI, lifestyle only explains 3.4% of its variation. Monastic females also show higher 6- and 4-biomarker SFI averages than nonmonastic females; however, as already observed, age, not lifestyle (monastic or nonmonastic) is the significant factor (Table 18).

## Discussion

### Skeletal frailty in monastic and nonmonastic Medieval samples

Results from ANOVA and ANCOVA demonstrate variable patterns of skeletal frailty between and within monastic and nonmonastic Medieval London samples. Overall, SFIs (2 to 11 biomarkers) in monastic skeletons are significantly higher, or approaching statistical significance (13-biomarker SFI), than their nonmonastic counterparts (Table 11). Although this disparity is explained partly by the greater representation of monastic individuals in older age categories, monastic and nonmonastic lifestyles also contribute significantly to this overall difference.

When intrasexual comparisons between monastic and nonmonastic contexts are considered, there are apparent differences within males and females. Among males, lifestyle and age both influence differences in SFI distributions between monastic and nonmonastic lifestyle (Table 14). By contrast, lifestyle, not age, is the sole significant influence on differences between frailty in female monastic and nonmonastic groups (Table 14). Discrete differences between females in monastic and nonmonastic contexts may reflect more definitive class distinctions between these groups. Monastic burial grounds, for example, which allowed for the interment of some lay persons, only included the most elite and privileged of their community. Therefore, the monastic cemeteries of Bermondsey Abbey and Merton Priory would have housed the remains of wealthy women rather than lower class working women associated with the nonmonastic community. These stark social divisions between representative females from the monastic and nonmonastic cemeteries would likely result in differential workloads and access to food resources and medical care, factors which influence somatic frailty [49, 51]. What is anomalous to this situation, however, is the higher frailty scores in the presumably elite monastically-associated females than the lower status nonmonastic females. Although physiological stress does not always correlate with SES groups [65, 66], this anomaly is explained likely by the few females in this sample (N_total_ = 23), and their unequal age distribution, within and outside the monastic setting. The contribution of age disparities to female frailty underscores this effect.

Monastic males show higher frailty than nonmonastic males (Table 14). These discrepancies reflect age and lifestyle differences. Our SFIs are composites of cumulative, irreversible biomarkers of frailty, thereby producing an inherent correlation with age. A greater proportion of monastic males are assigned to ages over 36 years than nonmonastic males (Table 6). Older age demographics reflect higher frailty. Regardless, ascribed monastic or nonmonastic lifestyles also contribute to frailty differences, with the former exhibiting higher frailty (Table 11). Monastic environments and lifestyles varied from those of the surrounding lay community. Bermondsey Abbey and Merton Priory were prominent manorial estates maintained through aristocratic and royal grants and donations. Zooarchaeological and archaeobotanical remains from Merton Priory provide evidence of the richness and variety of residents' diets consumed therein [53]. Medieval monastic life generally included reliable and better access to foodstuffs compared to the general population. Nonetheless, doctrines of monastic orders called for physical as well as spiritual health, and all residents participated in productive activities and daily chores [67]. Better access by monastic-living males to economic, nutritional, and medicinal resources arguably contributed to their longer lifespans and lower risk of mortality [48]. However, following monastic life and work conditions over these additional years of life, they incurred greater cumulative frailty.

### Consistency of the SFI

An inherent aspect of the 13-biomarker SFI proposed earlier is its inclusion of only individuals for whom a complete skeleton is preserved. This limits sample size available for study [1]. To aid in ameliorating this problem, we constructed and compared nonmetric SFIs of 2 to 11 skeletal biomarkers. We then compared these to our original 13-biomarker SFI to determine the influences of independent measures (lifestyle, age, and sex) in explaining variation in and distributions of SFIs constructed using fewer biomarkers. Using Spearman’s correlation coefficients, we compared the 10 nonmetric SFIs to results using the 13-biomarker SFI reported previously. Spearman’s correlations between the 13-biomarker SFI and nonmetric iterations all were statistically significant (Table 10), with a correlation range from 0.852 to 0.998 for indices including 6- to 11-biomarkers and a lower range, 0.645 to 0.818 for indices including 2- to 5-biomarkers with the 13-biomarker SFI. We used this 4% drop in association as a natural point to inform our further analyses. We determined a 6-biomarker SFI provides an estimate of skeletal frailty comparable to the original, metric SFI. Thus, the 6-biomarker should allow a similar, but not equivalent estimate of skeletal frailty when fewer biomarkers are available.

Based on the 13-biomarker SFI, frailty varied significantly by monastic and nonmonastic lifestyle, age category, and sex. Intersexual differences in frailty within the 134-individual Medieval sample, and intrasexual differences between monastic and nonmonastic settings are observed. Applying SFIs constructed of nonmetric biomarkers only, observed differences in SFIs by lifestyle, age, and sex remain significant, but become more pronounced, as explained variance increases (Tables 11–14). For lifestyle, ANCOVA results show that age and lifestyle (monastic/nonmonastic) both contribute significantly to frailty (Table 11). In particular, SFI constructs of 6- to 11-biomarkers yield significant differences between monastic and nonmonastic samples with lifestyle explaining 12–13% of total variation in assessed frailty.

Observed SFI distributions by age categories suggest a nuanced approach for applying SFIs. As with the 13-biomarker SFI, all nonmetric SFIs (sans the 2-biomarker SFI) differed significantly between age groups (Table 12). Post-hoc tests (Tukey’s HSD P-value 0.01) show these differences for 6- to 11-biomarker SFIs to be significant between age categories 1 and 4, and age categories 2 and 4 (S1 Table). For the 5- to 11-biomarker constructs, SFIs consistently increase from younger to older age categories, although, like the 13-biomarker SFI, a drop in average frailty occurred between the 18–25 year and the 26–35 year age categories. (Table 12). This higher frailty pattern among the youngest age category was initially interpreted as evidence of the osteological paradox, which suggests the frailest members of a population are most susceptible to death and therefore more likely to die at a younger age than their less frail counterparts [1, 27]. The new results from 5- to 11-biomarker SFI distributions provide additional support for this interpretation.

Nonmetric SFI results also show significant differences between sexes, reflective of the original 13-biomarker SFI results (Tables 13 and 14). SFI averages for males and females decrease from 3.20 to 2.46 and 2.87 to 2.22, respectively, between the 13- and 11-biomarker indices (Table 13). However, frailty differences are maintained using exclusively nonmetric biomarkers. Age contributes more significantly (P-values<0.05) to these differences for all nonmetric SFIs (except the 2-biomarker SFIs) than does sex (P-values≥0.05). The age distribution of males is older than females in this sample, accounting for this disparity in SFIs rather than a sex-related difference (Fig 2). The disproportionate sample size between sexes, especially among the monastic sample (an artifact of monastic burial traditions), presents an inherent sampling bias that may impact observed intersexual frailty differences (Fig 1).

While results using ANCOVA for the 6-biomarker and 4-biomarker SFIs compare with the 13-biomarker SFI results for intersexual variation, intrasexual differences between monastic and nonmonastic groups are significant for the reduced SFIs. Monastic males and females exhibit significantly higher skeletal frailty than nonmonastic males and females (Table 18). These results contrast with 13-biomarker SFI findings, although they align with the nonmetric 6- to 11-biomarker SFI and results from ANCOVA (Table 14).

Overall, nonmetric SFIs with 6 or more biomarkers indicate similar associations of frailty as observed with the original 13-biomarker SFI (Tables 11–14). Significant differences in SFI distributions by lifestyle, age, sex, and sex-lifestyle are retained when nonmetric SFIs of 6 or more biomarkers are used. However, despite similar results based on significance levels, explained variance in nonmonastic SFIs is lower. For example, when considering how lifestyle affects frailty differences between monastic and nonmonastic groups, lifestyle explains 5% of variation in the 13-biomarker SFIs; lifestyle explains twice as much variation, 12.1% to 13.5%, using the 6- to 11-biomarker SFIs (Table 11). Similar discrepancies occur for age and sex. Age explains only 4% of variation in the 13-biomarker SFI, but 8.2%-9.1% of variation in the 6- through 11- biomarker indices. Similarly, sex accounts for only 3.1% of total variation in the 13-biomarker SFI, but 7.5–9.1% in the 6- to 11 biomarker SFIs (Table 13). One possible reason why sex explains less variance in the 13-biomarker index than the nonmetric indices may be the similarity of maximal femoral lengths and femoral head diameters within sexes between monastic and nonmonastic groups. Student’s t-tests of femoral lengths and femoral head diameter between monastic and nonmonastic samples show no significant differences among males (P-value_femlgth_ = 0.08, P-value_femhead_ = 0.06) or among females (P-value_femlgth_ = 0.75, P-value_femhead_ = 0.86). If these two Medieval London samples are genetically similar, this congruency could affect growth trajectories; although stature is a polygenic trait influenced by exogenous factors, maximum and minimum statures may be relatively genetically stable. Any growth deficits within these Medieval individuals, which fall within the range of normal variation of this London population, therefore, may not appear as childhood growth perturbations. Another suggestion for this discrepancy in explained variation between 13-biomarker and nonmetric biomarker SFIs is that the former captures to a greater degree later life insults to the skeleton that may be equally affecting both settings that are not due to either age or sex, while SFIs without early markers are more apt to show the signs of age and sex-based influences.

### Evaluating 6- and 4-biomarker SFIs in larger samples

Metric biomarkers may not always be assayable on skeletons. In such situations, SFIs based solely on nonmetric biomarkers or composed of only four biomarkers may produce results similar to a 13-biomarker metric SFI. We selected the 6-biomarker SFI from the suite of 10 SFI iterations because it not only yielded a comparable SFI distribution (Spearman’s rho = 0.852) but also demonstrated similar P-values and explained variance from ANOVA/ANCOVA tests for lifestyle, age, and sex covariates as the 13-biomarker SFI (Tables 11–14). Subsequently, we developed a 4-biomarker SFI composed of variables from each of four “health” categories and applied this and the 6-biomarker SFI to a larger Medieval sample. Removal of metric variables increased the sample size to 344 monastic and 123 nonmonastic individuals (N_total_ = 517).

Both the 4- and 6-biomarker SFI distributions for monastic and nonmonastic samples indicate significantly higher frailty in the former group (Table 15). However, whereas age and lifestyle influence this difference in the 6-SFI distributions, lifestyle does not contribute significantly to differences in 4-biomarker SFIs. In general, age significantly affects both the 4- and 6-biomarker SFIs: as age categories become higher, average frailty increases (Table 16). However, post-hoc Tukey’s HSD tests show that this difference is significant only between older and younger age categories for the 6-biomarker SFI and between only the first and third age categories for the 4-biomarker SFI (S1 Table). Regarding intersexual differences in SFI, age—not sex—contributes significantly to both nonmetric SFIs. When comparing these results with those from the 13-biomarker SFI study, there are multiple commonalities (Table 17). ANOVA/ANCOVA of the 6-biomarker SFI yield similar statistical results as the 13-biomarker and other nonmetric SFIs: significant differences in SFI are observed between monastic and nonmonastic samples and age categories, but not within or between the sexes when controlled for age (Tables 17 and 18). By contrast, the parsimonious 4-biomarker SFI results only demonstrate significant changes in SFIs between age categories 1 and 3 (S2 Table).

Based upon results using ANOVA/ANCOVA, age is associated significantly with differences between monastic-nonmonastic groups and sex subgroups in the 4-biomarker SFIs (Tables 17 and 18; S2 Table). The 4-biomarker SFI includes LEH, periosteal lesion, osteoarthritis (OA), and trauma. Since trauma and OA often are associated directly with age, they likely contribute to age-related influences on frailty. The 6-biomarker index includes two additional biomarkers, PD and IVD, which correlate significantly with age. Although PD is indicative of infection and IVD indicative of wear to the vertebrae, these conditions occur at higher frequencies in older age categories. Despite having more age-dependent variables than the 4-biomarker SFI, frailty differences using the 6-biomarker SFIs are explained by other contributing factors (e.g., lifestyle), not just age. These results support previous research in living populations suggesting frailty is not exclusively an age-dependent phenotype; rather, frailty is influenced by numerous genetic, socioeconomic, cultural, and political factors [35, 38, 68].

### Selecting and applying 13-biomarker SFIs or nonmetric SFIs

By examining 10 different SFIs (including 2 to 11 biomarkers) and applying these to Medieval London monastic and nonmonastic samples, we were able to determine that at least six biomarkers likely are required to generate SFIs to yield results for explained variation by lifestyle, age, and sex and frailty scores consistent with the 13-biomarker index in these Medieval London samples. When indices include five or fewer biomarkers, total variation is less well explained by available independent variables (lifestyle, age, and sex), and multiple non-significant results are observed. For this Medieval sample, these analyses indicate SFIs including a minimum of six biomarkers are useful for assessing skeletal frailty without significant loss of explanatory power compared to the initial 13-biomarker construct. However, it is advised that metric biomarkers be retained whenever possible as they are robust indicators of childhood stress and likely reflect adult life stressors. Biomarkers of physical activity, osteoarthritis and rotator cuff disease, result from cumulative, irreversible processes that often are associated with age [16]. Similarly, trauma and the sequelae of infections likely accumulate with age in those who survive. Thus, increases in skeletal frailty with age are expected and reflect such chronic processes. By contrast, stature and skeletal robusticity stabilize early in adulthood. Latent and lasting effects of childhood stressors on growth and development may lead to increased susceptibility to pathogenic, nutritional, and/or social insults during later years of life [25, 69]. For example, individuals affected by compromised growth exhibit a higher incidence of cardiovascular disease than other members of their age categories [70]. We observed such a pattern in the monastic and nonmonastic samples for the 13-biomarker SFI, which was 3.00 in the youngest age category, but only 2.62 in the following age category. This level of detailed results using SFIs with stature and robusticity biomarkers included still is observed when reduced-variable (6- to 11-biomarker) SFIs are applied, illustrating the efficacy of nonmetric biomarkers for assessing nuances in skeletal frailty. Nonetheless, whenever sample size is adequately reflective of the population, we recommend using the full set of 13 biomarkers to construct a skeletal frailty index. Given restrictions on data availability, reduced nonmetric SFI constructs may be more useful as they also vary significantly with lifestyle, age, and sex across skeletal samples and provide an avenue for comparative analyses across biological and sociocultural categories.

When constructing and applying SFIs to bioarchaeological samples for comparative analysis, preservation and site-specific paleopathological and paleoepidemiological profiles must be considered. We do not mandate that bioarchaeologists apply our specific 13-biomarker SFI or 6-biomarker SFI to populations other than Medieval London, especially if these SFIs reduce representative sample size and frailty information. Rather, we recommend that bioarchaeologists construct a site (or cross-site) specific metric and nonmetric SFI and subsequently remove biomarkers, in the iterative way demonstrated, to increase sample size and statistical power. As long as researchers justify and explain their site-specific SFIs, and how these indices contribute information about individual frailty, SFIs need not be identically constructed. We argue that the greatest strength of the SFI is its mutable design to first maximize frailty information and second increase sample size.

## Conclusions

This paper addresses a possible shortcoming of the original 13-biomarker SFI proposed by Marklein and colleagues [1], namely, that it leads to significantly reduced sample sizes. Bearing in mind the inevitability of poor preservation in human osteological assemblages, we tested how nonmetric SFIs, comprised of 2 to 11 biomarkers, would compare to the original 13-biomarker SFI results in terms of average SFI scores, significance, and explained variation by ascribed lifestyle, age, and sex. While the 13-biomarker SFI still provides the most informative results, especially when considering early adulthood frailty, other SFIs containing 6 to 11 biomarkers yield similar results to the 13-biomarker SFI distributions between Medieval monastic and nonmonastic samples. Although SFIs with more biomarkers offer a more complete picture of frailty, our findings suggest that bioarchaeologists may usefully apply 6-biomarker SFIs to their research and achieve SFI results comparable to the 13-biomarker SFI, with the added advantage of larger sample sizes, and, consequently, greater statistical power.

We developed the skeletal frailty index as a tool to complement current methods, such as gross prevalence counts and hazard analyses, for evaluating skeletal “frailty” and “health”. We propose two major strengths for the SFI. First, it is based upon methods and biomarkers used to assess the frailty phenotype in living human samples as opposed to measuring disease or health status [2, 3, 7]. Second, the SFI is widely applicable and versatile in its construction. Based upon results presented here and reported by Marklein and colleagues [1], the 13-biomarker SFI is a robust measure of skeletal frailty and reflective of frailty indices used among the living. In addition, indices constructed using as few as 6 biomarkers appear to be useful for illustrating how age, sex, and lifestyles attributed to ancient skeletons may be predictive of their phenotypic frailty during life. As metric biomarkers may not be assayable in many skeletal collections, SFIs based only on nonmetric biomarkers, and even one composed of only four biomarkers, may produce results reflective of, but not identical to nor as specific as, the 13-biomarker SFI. The utility of SFIs constructed using fewer biomarkers makes this method applicable to more skeletal collections and increases sample sizes available for analyses.

## Supporting information

S1 Table Tukey’s HSD post-hoc test for SFI and age categories (n = 134). Significance (*) at p<0.05.
(DOCX)

S2 Table Tukey’s HSD post-hoc test for SFI and age categories (N = 517). Significance (*) at p<0.05.
(DOCX)

## References

[1] Marklein KE, Leahy RE, Crews DE. In sickness and in death: assessing frailty in human skeletal remains. American Journal of Physical Anthropology. 2016. 10.1002/ajpa.23019 27312014

[2] Fried LP, Ferrucci L, Darer J, Williamson JD, Anderson G. Untangling the concepts of disability, frailty, and comorbidity: Implications for improved targeting and care. The Journals of Gerontology Series A: Biological Sciences and Medical Sciences. 2004;59(3):M255–63. 10.1093/gerona/59.3.m255 15031310

[3] Crews DE. Evolutionary Perspectives on Human Longevity and Frailty In: Carey JR, Robine J-M, Michel JP, Christen Y, editors. Longevity and Frailty. Research and Perspectives in Longevity. Berlin: Springer-Verlag 2005 p. 57–65.

[4] Walston JD. Biological Markers and the Molecular Biology of Frailty In: Carey JR, Robine J-M, Michel JP, Christen Y, editors. Longevity and Frailty. Research and Perspectives in Longevity. Berlin: Springer-Verlag 2005 p. 83–90.

[5] Dent E, Chapman I, Howell S, Piantadosi C, Visvanathan R. Frailty and functional decline indices predict poor outcomes in hospitalised older people. Age and Ageing. 2013:1–7. 10.1093/ageing/aft181 24257468

[6] Dent E, Hoogendijk EO. Psychosocial factors modify the association of frailty with adverse outcomes: a prospective study of hospitalised older people. BMC Geriatrics. 2014;14:108–15. 10.1186/1471-2318-14-108 25262425 PMC4190287

[7] Kim S, Jazwinski SM. Quantitative measures of healthy aging and biological age. Healthy Aging Research. 2015;4:26 10.12715/har.2015.4.26 26005669 PMC4440677

[8] Buikstra JE. Biocultural Dimensions of Archaeological Study: A Regional Perspective In: Blakely RL, editor. Biocultural Adaptation in Prehistoric America. Athens: University of Georgia Press; 1977 p. 67–84.

[9] Cohen MN, Armelagos GJ, editors. Paleopathology at the origins of agriculture. New York: Academic Press, Inc 1984.

[10] Martin DL, Harrod RP, Pérez VR. Bioarchaeology; an integrated approach to working with human remains. New York: Springer 2013.

[11] Larsen CS. Bioarchaeology: Interpreting Behavior from the Human Skeleton. 1st ed New York: Cambridge University Press; 1997.

[12] Larsen CS. Bioarchaeology: Interpreting Behavior from the Human Skeleton 2nd ed Cambridge: Cambridge University Press; 2015.

[13] Goodman AH, Martin DL. Reconstructing health profiles from skeletal remains In: Steckel RH, Rose JC, editors. The Backbone of History: Health and Nutrition in the Western Hemisphere. New York: Cambridge University Press; 2002 p. 11–61.

[14] Ortner DJ. Identification of Pathological Conditions in Human Skeletal Remains. New York: Academic Press; 2003.

[15] Roberts C, Manchester K. The Archaeology of Disease. Ithaca: Cornell University Press; 2005.

[16] Waldron T. Palaeopathology. Cambridge: Cambridge University Press; 2009.

[17] Fried LP, Xue QL, Cappola AR, Ferrucci L, Chaves P, Varadhan R, et al Nonlinear multisystem physiological dysregulation associated with frailty in older women: implications for etiology and treatment. Journal of Gerontology. 2009;9:1049–57. 10.1093/gerona/glp076 PMC2737590 19567825

[18] Rockwood K, Mogilner A, Mitnitski A. Changes with age in the distribution of a frailty index. Mechanisms of Ageing and Development. 2004;125(7):517–9. 10.1016/j.mad.2004.05.003 15246748

[19] Basic D, Shanley C. Frailty in an Older Inpatient Population: Using the Clinical Frailty Scale to Predict Patient Outcomes. Journal of Aging and Health. 2014:1–14. 10.1177/0898264314558202 25414168

[20] Evans SJ, Sayers M, Mitnitski A, Rockwood K. The risk of adverse outcomes in hospitalized older patients in relation to a frailty index based on a comprehensive geriatric assessment. Age and Ageing. 2014;43:127–32. 10.1093/ageing/aft156 24171946

[21] Vidovič M, Sharron G, Crews DE. Correlates of frailty among aging residents of pper Selška Valley Villages under Ratitovec Mountain. Collegium Antropologicum. 2015;39(2):297–306. 26753446

[22] Chamberlain AM, St. Sauver JL, Jacobson DJ, Manemann SM, Fan C, Roger VL, et al Social and behavioural factors associated with frailty trajectories in a population-based cohort of older adults. BMJ Open. 2016;6:10.1136/bmjopen-2016-011410 PMC4885446 27235302

[23] Usher BM. A multistate model for health and mortality in paleodemography: TIrup cemetery [PhD Dissertation]. State College: Pennsylvania State University; 2000.

[24] DeWitte SN, Wood JW. Selectivity of Black Death mortality with respect to preexisting health. Proceedings of the National Academy of Sciences USA. 2008;105:1436–41. 10.1073/pnas.0705460105 PMC2234162 18227518

[25] DeWitte SN, Hughes-Morey G. Stature and frailty during the Black Death: the effect of stature on risks of epidemic mortality in London, A.D. 1348–1350. Journal of Archaeological Research. 2012;39:1412–9. 10.1016/j.jas.2012.01.019 PMC3868458 24363485

[26] Crimmins EM. Trends in the health of the elderly. Annual Review of Public Health. 2004;25:79–98. 10.1146/annurev.publhealth.25.102802.124401 15015913

[27] Wood JW, Milner GR, Harpending HC, Weiss KM. The Osteology Paradox: problems of inferring health from skeletal samples. Current Anthropolgy. 1992;33(4): 343–70.

[28] Crimmins EM, Kim JK, Sole-Auro A. Gender differences in health: results from SHARE, ELSA, and HRS. European Journal of Public Health. 2010;21(1):81–91. 10.1093/eurpub/ckq022 20237171 PMC3023013

[29] Oksuzyan A, Juel K, Vaupel JW, Christensen K. Men: good health and high mortality. Sex differences in health and aging. Aging Clinical and Experimental Research. 2008;20(2):91–102. 18431075 10.1007/bf03324754 PMC3629373

[30] Šlaus M. Biocultural analysis of sex differences in mortality profiles and stress levels in the late medieval population from Nova Rača, Croatia. American Journal of Physical Anthropology. 2000;111(2):193–209. 10640947 10.1002/(SICI)1096-8644(200002)111:2<193::AID-AJPA6>3.0.CO;2-0

[31] Trovato F, Lalu NM. Narrowing sex differentials in life expectancy in the industrialized world: Early 1970’s to early 1990’s. Social Biology. 1996;43:20–37. 8909108 10.1080/19485565.1996.9988911

[32] Waldron I. What do we know about causes of sex differences in mortality? A review of the literature. Population Bulletin of the United Nations. 1985;18:59–76. 12314310

[33] Buikstra JE, Ubelaker DH. Standards for Data Collection from Human Skeletal Remains. Fayetteville: Arkansas Archeological Society 1994.

[34] Steckel RH, Larsen CS, Sciulli PW, Walker PL. The Global History of Health Project Data Collection Codebook. 2005. http://global.sbs.ohio-state.edu/new_docs/Codebook-01-24-11-em.pdf [Accessed 1st July 2016].

[35] Bird CE, Seeman T, Escarce JJ, Basurto-Dávila R, Finch BK, Dubowitz T, Heron M, et al Neighbourhood socioeconomic status and biological 'wear and tear' in a nationally representative sample of US adults. Journal of Epidemiology and Community Health. 2010;64(10):860–5. 10.1136/jech.2008.084814 19759056 PMC3432399

[36] Crimmins EM, Kim JK, Seeman TE. Poverty and biological risk: the earlier "aging" of the poor. Journal of Gerontology. 2009;64A(2):286–92. 10.1093/gerona/gln010 PMC2655037 19196637

[37] Robertson T, Batty GD, Der G, Fenton C, Shiels PG, Benzeval M. Is socioeconomic status associated with biological aging as measured by telomere length? Epidemiologic reviews. 2012:mxs1–14. 10.1093/epirev/mxs001 PMC3578449 23258416

[38] Robertson T, Popham F, Benzeval M. Socioeconomic position across the lifecourse & allostatic load: date from the West of Scotland Twenty-07 cohort study. BMC Public Health. 2014;14:184–92. 24555560 10.1186/1471-2458-14-184 PMC3942053

[39] Szanton SL, Gill JM, Allen JK. Allostatic Load: A mechanism of socioeconomic health disparities? Biological Research for Nursing. 2005;7(1):7–15. 53 10.1177/1099800405278216 15919999 PMC2874580

[40] DeWitte SN. Differential survival among individuals with active and healed periosteal new bone formation. International Journal of Paleopathology. 2014;7:38–44. 29539489 10.1016/j.ijpp.2014.06.001

[41] Bennike P, Lewis ME, Schutkowski H, Valentin F. Comparison of child morbidity in two contrasting medieval cemeteries from Denmark. American Journal of Physical Anthropology. 2005;128(4):734–46. 10.1002/ajpa.20233 16044468

[42] Seeman E. The periosteum—a surface for all seasons. Osteoporosis International. 2007;18(2):123–8. 10.1007/s00198-006-0296-6 17180552

[43] Weston DA. Investigating the specificity of periosteal reactions in pathology museum specimens. American Journal of Physical Anthropology. 2012;137:48–59. 10.1002/ajpa.20839 18398845

[44] DeWitte SN, Bekvalac J. The association between periodontal disease lesions in the St. Mary Graces cemetery, London, England AD 1350–1538. American Journal of Physical Anthropology. 2011;152:322–32. 10.1002/ajpa.21622 21997205

[45] Milner GR, Anderson E, Smith VG. Warfare in Late Prehistoric West-Central Illinois. American Antiquity. 1991;56(4):581–603.

[46] Walker PL. A bioarchaeological perspective on the history of violence. Annual review of Anthropology. 2001;30:573–96.

[47] Goldman N, Glei DA, Seplaki C, Liu IW, Weinstein M. Perceived stress and physiological dysregulation in older adults. 2005;8:95–105. 10.1080/10253890500141905 16019601

[48] DeWitte SN, Boulware JC, Redfern RC. Medieval monastic mortality: hazard analysis of mortality differences between monastic and nonmonastic cemeteries in England. American Journal of Physical Anthropology. 2013;152:322–32. 10.1002/ajpa.22350 24014273

[49] Mollat M. The poor in the Middle Ages: an essay in social history. New Haven: Yale University Press 1986.

[50] Pounds N. The Medieval City. Wesport: Greenwood Press 2005.

[51] Amt E, editor. Women’s Lives in Medieval Europe: A Sourcebook. New York: Routledge 1993.

[52] Powers N, editor. Human osteology method statement. London: Museum of London; 2012.

[53] Miller P, Saxby D. The Augustinian priory of St. Mary Merton, Surrey. Excavations 1976–1990. London: Museum of London 2007.

[54] WORD Database, Museum of London. https://www.museumoflondon.org.uk/collections/other-collection-databases-and-libraries/centre-human-bioarchaeology/osteological-database [Accessed 20th June 2016].

[55] Knowles D, Hadcock RN. Medieval religious houses, England and Wales. London: Longman 1971.

[56] Dyson T, Dyson T, Samuel M, Steele A, Steele A. The Cluniac priory and abbey of St. Saviour, Bermondsey, Surrey: excavations 1984–95. London: Museum of London Archaeology 2011.

[57] Bowsher D, Dyson T, Holder N, Howell I. The London Guildhall: an Archaeological History of a Neighbourhood from Early Medieval to Modern Times. London: Museum of London Archaeology 2007.

[58] Thomas C, Sloane B, Phillpotts C, editors. Excavations at the priory and hospital of St. Mary Spital. London: Museum of London 1997.

[59] Grainger I, Phillpotts C. The Cistercian Abbey of St. Mary Graces, East Smithfield, London MOLA monograph 44. London: Museum of London Archaeology 2011.

[60] Miles A, White W, Tankard D. Burial at the site of the parish church of St. Benet Sherehog before and after the Great Fire: excavations at 1 Poultry, City of London. London: Museum of London Archaeology Service 2008.

[61] Goodman AH, Martin DL, Armelagos GJ. Indications of stress from bone and teeth In: Cohen MN, Armelagos GJ, editors. Paleopathology at the Origins of Agriculture. Orlando: Academic Press; 1984 p. 13–44.

[62] Löe H, Ånerud Å, Boysen H. The natural history of periodontal disease in man: prevalence, severity, and extent of gingival recession. Journal of Periodontology. 1992;63(6):489–95. 1625148 10.1902/jop.1992.63.6.489

[63] Smith ML, Mott A, Marques CPC, Maor Y, de Andrade MS, Rodrigues VP, et al Position paper: epidemiology of periodontal diseases. Journal of Periodontology. 2005;76(8):14606–1419. 10.1902/jop.2005.76.8.1406 16101377

[64] Grauer AL. Patterns of anemia and infection from medieval York, England. American Journal of Physical Anthropology. 1993;91(2):203–13. 8317561 10.1002/ajpa.1330910206

[65] Sullivan A. Reconstructing relationships among mortality, status, and gender at the Medieval Gilbertine Priory of St. Andrew, Fishergate, York. American Journal of Physical Anthropology. 2004;124(4);330–45. 15252861 10.1002/ajpa.10271

[66] DeWitte SN, Hughes-Morey G, Bekvalac J, Karsten J. Wealth, health and frailty in industrial-era London. Annals of Human Biology. 2016;43(3):241–54. 10.3109/03014460.2015.1020873 26073638

[67] Lawrence CH. Medieval monasticism: forms of religious life in Western Europe in the Middle Ages. London: Longman Group Ltd; 1984.

[68] Dowd JB, Simanek AM, Aiello AE. Socio-economic status, cortisol and allostatis load: a review of the literature. International Journal of Epidemiology. 2009;1–13. 10.1093/ije/dyp277 PMC2755130 19720725

[69] Yaussy SL, DeWitte SN, Redfern RC. Frailty and Famine: patterns of mortality and physiological stress among victims of famine in Medieval London. American Journal of Physical Anthropology. 2016;160(2):272–83. 10.1002/ajpa.22954 26854255

[70] Hebert PR, Rich-Edwards JW, Manson JE, Ridker PM, Cook NR, O'Connor GT, et al Height and incidence of cardiovascular disease in male physicians. Circulation. 1993;88(4):1437–43. 8403290 10.1161/01.cir.88.4.1437

[71] Hillson S. Dental anthropology, 2nd edition Cambridge: Cambridge University Press 1996.

[72] Roberts J, Connell B. Palaeopathology In: Brickley M, McKinley JI, editors. Guidelines to the Standards for Recording Human Remains Reading. BABAO; 2004 p. 34–39.

[73] Brothwell DR. Digging up bones: the excavation, treatment and study of human skeletal remains, 3rd edition London: Cornell University Press 1981.

[74] Stuart-Macadam PL. Anemia in Roman Britain: Poundbury Camp In: Bush H, Zvelebil M, editors. Health in past societies: biocultural interpretations of human skeletal remains in archaeological contexts. Oxford: BAR International Series 1991.

[75] Brickley M, Mays S, Ives R. An investigation of skeletal indicators of vitamin D deficiency in adults: effective markers for interpreting past living conditions and pollution levels in 18^th^ and 19^th^ century Birmingham, England. American Journal of Physical Anthropology. 2007;123(1):67–79. 10.1002/ajpa.20491 17078033

[76] Resnick D, editor. Diagnosis of Bone and Joint Disorders, 4th edition Philadelphia: Saunders 2002.

[77] Aufderheide AC, Rodriguez-Martin C. 19998. The Cambridge Encyclopaedia of Human Palaeopathology. Cambridge: Cambridge University Press 1998.

[78] Rogers J, Waldron T. A field guide to joint disease in archaeology. New York: John Wiley & Sons, Inc 1995.

[79] Roberts CA, Trauma in biocultural perspective: past, present, and future work in Britain In: Cox M, Mays S, editors. Human osteology in archaeology and forensic sicence. London: Greenwich Medieval Media; 2000 p. 337–56.

