# Supplementary material for: Frail or hale: Skeletal frailty indices in Medieval London skeletons
Source: PLoS One. 2017 May 3;12(5):e0176025. doi: 10.1371/journal.pone.0176025 (PMC5415061; doi:10.1371/journal.pone.0176025)
Supplement: S1 Table — Significance (*) at p<0.05. (DOCX) [file pone.0176025.s001.docx]

**S1 Table**. Tukey’s HSD post-hoc test for SFI and age categories (n=134). Significance (*) at p<0.05.

| Age Category | Age Category | 13 SFI | 11-SFI | 10-SFI | 9-SFI | 8-SFI | 7-SFI | 6-SFI | 5-SFI | 4-SFI | 3-SFI | 2-SFI |
| --- | --- | --- | --- | --- | --- | --- | --- | --- | --- | --- | --- | --- |
| 1 | 2 | 0.952 | 0.932 | 0.967 | 0.967 | 0.966 | 0.988 | 1.000 | 0.999 | 0.983 | 0.877 | 0.687 |
|  | 3 | 0.962 | 0.399 | 0.306 | 0.306 | 0.298 | 0.220 | 0.114 | 0.508 | 0.219 | 0.101 | 0.563 |
|  | 4 | 0.218 | 0.042* | 0.027* | 0.027* | 0.034* | 0.030* | 0.013* | 0.208 | 0.080 | 0.020 | 0.628 |
| 2 | 1 | 0.952 | 0.932 | 0.967 | 0.967 | 0.966 | 0.988 | 1.000 | 0.999 | 0.983 | 0.877 | 0.687 |
|  | 3 | 0.366 | 0.070 | 0.067 | 0.067 | 0.063 | 0.061 | 0.061 | 0.337 | 0.341 | 0.375 | 1.000 |
|  | 4 | 0.003* | 0.003* | 0.003* | 0.003* | 0.004* | 0.006* | 0.006* | 0.117 | 0.128 | 0.091 | 1.000 |
| 3 | 1 | 0.962 | 0.399 | 0.306 | 0.306 | 0.298 | 0.220 | 0.114 | 0.508 | 0.219 | 0.101 | 0.563 |
|  | 2 | 0.366 | 0.070 | 0.067 | 0.067 | 0.063 | 0.061 | 0.061 | 0.337 | 0.341 | 0.375 | 1.000 |
|  | 4 | 0.052 | 0.398 | 0.390 | 0.390 | 0.462 | 0.542 | 0.541 | 0.807 | 0.827 | 0.695 | 1.000 |
| 4 | 1 | 0.218 | 0.042* | 0.027* | 0.027* | 0.034* | 0.030* | 0.013* | 0.208 | 0.080 | 0.020 | 0.628 |
|  | 2 | 0.003* | 0.003* | 0.003* | 0.003* | 0.004* | 0.006* | 0.006* | 0.117 | 0.128 | 0.091 | 1.000 |
|  | 3 | 0.052 | 0.398 | 0.390 | 0.390 | 0.462 | 0.542 | 0.541 | 0.807 | 0.827 | 0.695 | 1.000 |
