# Supplementary material for: Frail or hale: Skeletal frailty indices in Medieval London skeletons
Source: PLoS One. 2017 May 3;12(5):e0176025. doi: 10.1371/journal.pone.0176025 (PMC5415061; doi:10.1371/journal.pone.0176025)
Supplement: S2 Table — Significance (*) at p<0.05. (DOCX) [file pone.0176025.s002.docx]

**S2 Table**. Tukey’s HSD post-hoc test for SFI and age categories (N=517). Significance (*) at p<0.05.

| Age Category | Age Category | 6-biomarker SFI | 4-biomarker SFI |
| --- | --- | --- | --- |
| 1 | 2 | 0.639 | 0.558 |
|  | 3 | 0.002* | 0.019* |
|  | 4 | 0.000* | 0.230 |
| 2 | 1 | 0.639 | 0.558 |
|  | 3 | 0.004* | 0.135 |
|  | 4 | 0.001* | 0.847 |
| 3 | 1 | 0.002* | 0.019* |
|  | 2 | 0.004* | 0.135 |
|  | 4 | 0.669 | 0.673 |
| 4 | 1 | 0.000* | 0.230 |
|  | 2 | 0.001* | 0.847 |
|  | 3 | 0.669 | 0.673 |
